# Psycho-physio-neurological correlates of qualitative attention, emotion and flow experiences in a close-to-real-life extreme sports situation: low- and high-altitude slackline walking

**DOI:** 10.7717/peerj.17743

**Published:** 2024-07-26

**Authors:** Marcelo Felipe de Sampaio Barros, Carlos Alberto Stefano Filho, Lucas Toffoli de Menezes, Fernando Manuel Araújo-Moreira, Luis Carlos Trevelin, Rafael Pimentel Maia, Rémi Radel, Gabriela Castellano

**Affiliations:** 1 Programa de Pós-graduação em Biotecnologia, Universidade Federal de São Carlos (UFSCar), São Carlos, São Paulo, Brazil; 2 Laboratoire LAMHESS, Université de Nice Sophia Antipolis, Nice, Côte d’Azur, France; 3 Neurophysics Group, Gleb Wataghin Institute of Physics, Universidade Estadual de Campinas, Campinas, São Paulo, Brazil; 4 Brazilian Institute of Neuroscience and Neurotechnology (BRAINN), Campinas, São Paulo, Brazil; 5 Programa de pós-graduação em Engenharia Nuclear, Instituto Militar de Engenharia/IME, Rio de Janeiro, Rio de Janeiro, Brazil; 6 Departamento de Computação, Universidade Federal de São Carlos (UFSCar), São Carlos, São Paulo, Brazil; 7 Department of Statistics, Institute of Mathematics, Statistics and Scientific Computing, Universidade Estadual de Campinas, Campinas, São Paulo, Brazil

**Keywords:** Extreme sports, Qualitative attention, Emotion, Flow experiences, Psychophysiology, EEG

## Abstract

It has been indicated that extreme sport activities result in a highly rewarding experience, despite also providing fear, stress and anxiety. Studies have related this experience to the concept of flow, a positive feeling that individuals undergo when they are completely immersed in an activity. However, little is known about the exact nature of these experiences, and, there are still no empirical results to characterize the brain dynamics during extreme sport practice. This work aimed at investigating changes in psychological responses while recording physiological (heart rate–HR, and breathing rate–BR) and neural (electroencephalographic–EEG) data of eight volunteers, during outdoors slackline walking in a mountainous environment at two different altitude conditions (1 m–low-walk– and 45 m–high-walk–from the ground). Low-walk showed a higher score on flow scale, while high-walk displayed a higher score in the negative affect aspects, which together point to some level of flow restriction during high-walk. The order of task performance was shown to be relevant for the physiological and neural variables. The brain behavior during flow, mainly considering attention networks, displayed the stimulus-driven ventral attention network–VAN, regionally prevailing (mainly at the frontal lobe), over the goal-directed dorsal attention network–DAN. Therefore, we suggest an interpretation of flow experiences as an opened attention to more changing details in the surroundings, *i.e*., configured as a ‘task-constantly-opened-to-subtle-information experience’, rather than a ‘task-focused experience’.

## Introduction

In the last decades, extreme sports which imply risk, such as rock-climbing in hazardous environments, mountaineering, base jumping, or other physical activities with odds of deadly falls, have become extremely popular (Brymer et al., 2020; Kerr & Houge Mackenzie, 2012; Lee et al., 2024; Pizam, Reichel & Uriely, 2001). While this practice has been traditionally associated with people with a high sensation-seeking personality (*e.g*., Pizam, Reichel & Uriely, 2001), recent conceptualization indicates that participation in these activities would extend beyond this restrictive profile by satisfying many different motives (Clough et al., 2016; Kerr & Houge Mackenzie, 2012) and by providing a highly rewarding experience (Immonen et al., 2017). Because high-risk sports typically also imply fear, stress, and anxiety (Hare, Wetherell & Smith, 2013; Monasterio et al., 2016; Woodman et al., 2009), the positive experience must be very intense to prevail over these negative emotions. However, little is known about the exact nature of these experiences.

Studies investigating psychophysiological changes associated with extreme sport have indicated that some alterations are triggered before practice. Marked increases in self-reported anxiety and cortisol are typically seen at the resting period preceding the practice (Chatterton et al., 1997; Hare, Wetherell & Smith, 2013; Mujica-Parodi et al., 2014), peaking during practice (Hare, Wetherell & Smith, 2013; Mujica-Parodi et al., 2014). Interestingly, the amplitude of the cortisol response in skydiving was found to be highly correlated with amygdala reactivity in a separate threat perception task (Mujica-Parodi et al., 2014). Besides the hypothalamic–pituitary–adrenal axis activation, the autonomous nervous system is also importantly affected during extreme sports practice. Physiological signals (usually accessed by wearable devices), can reflect people’s emotions. Indeed, emotions influence the activity of the autonomous nervous system (ANS), which in turn regulates various body parameters such as heart rate, respiration and temperature pattern variations. Besides of the wearable devices, contactless technologies, such thermal infrared (IR) imaging based on machine learning approaches are starting to be used for the assessment of emotions (Filippini et al., 2022). Skydiving jump leads to increased sympathetic activity as indexed by higher salivary alpha-amylase (Chatterton et al., 1997) or heart rate (HR) (Allison et al., 2012), which suggests a state of hyperarousal (Sterlini & Bryant, 2002). However, if the activation of the sympathetic system can be part of the stress response, results showing dissociation of the sympathetic arousal from cortisol reactivity (Monasterio et al., 2016) suggest the involvement of other factors, such as emotional excitement (Shiota et al., 2011), or the activation of the brain reward system (Critchley, Mathias & Dolan, 2001). Supporting this idea, it has been shown that risk sensation seeking is related to dopaminergic activity (German & Bowden, 1974), and that extreme sports could lead to similar effects to those of drugs that stimulate the reward system, contributing to anhedonia (Franken, Zijlstra & Muris, 2006) and to a state of dependency (Partington, Partington & Olivier, 2009). It is interesting to note that the sympathetic activation during extreme sport practice does not necessarily coincide with parasympathetic deactivation, but, in contrast, the activations of the two autonomous nervous branches appear to occur in a concurrent way at the same time. Specifically, skydiving has been found to elevate both HR, a sympathetic signal, and heart rate variability (HRV), a parasympathetic acting indication (Allison et al., 2012). Parasympathetic activation should help individuals to control typical anxiety effects during extreme sports. Co-activation of the two autonomous nervous branches would thus lead to a control sensation state with high awake and elevated calmness (Engelbregt et al., 2022) often bringing experiences of ‘relaxed subjective tension’ in action during risk situations. Such simultaneous sympathetic/parasympathetic co-activation has been related to the concept of flow experiences (Harmat et al., 2015; Ullén et al., 2010), a positive feeling that individuals experience when they are completely immersed in an activity (Polechoński et al., 2024; Csikszentmihalyi, 2014). Recent conceptualization of flow describes it as a state of effortless attention (Bruya, 2010), which could suggest flow as ‘relaxed high attention’ experiences. Many accounts have proposed that extreme sports would represent a perfect situation to induce flow (Jackson & Csikszentmihalyi, 1999; Pain & Pain, 2005). Flow states occur suddenly at critical time points (Weber & Fisher, 2020). Surgery and mountain climbing are highly critical tasks, which are more often reported to result in intense, ecstatic flow experiences, whereas yet absorbing but less critical tasks such as reading and video games, have less intense flow feelings (Gold & Ciorciari, 2020). Nevertheless, despite Gold & Ciorciari’s (2020) opinion in their article, however, a person can experience intense flow also during a video game playing (*e.g*., an expert gamer playing a new game). This will depends on the person and on their activity. The fact is that extreme sports provide alternative perceptions of space, time, and clarity or an augmented state of sensual awareness, that seem to facilitate flow, and, when in turn that happens, it tends to enhance performers’ ability to act (Brymer & Schweitzer, 2017). The study of the psychophysiological changes associated with the practice of extreme sports, reflecting on autonomic nervous systems behavior, suggests that this practice leads to a unique and complex experience that combines aspects of stress, anxiety, arousal, excitement, reward, and concentration (emotion and attention qualities), that can culminate in flow experiences. Since these elements recruit different neural networks, which are sometimes seem to work more in opposition than in congruence (Hermans et al., 2014), it would be interesting to better examine the neural patterns occurring during extreme sport performance. Studies about emotion (Doufesh et al., 2014), attention (Jäncke, Leipold & Burkhard, 2018), or both (Engelbregt et al., 2022), have shown autonomic nervous system body responses with relation to electroencephalography (EEG) measures.

Several studies have suggested that emotional perception tends to be brain lateralized by valence (Mańkowska et al., 2018; Martin & Altarriba, 2017). The hemispheric laterality valence hypothesis (Hellige, 1993) proposes that the right hemisphere is dominant for processing negative, unpleasant emotions, with the left hemisphere being dominant for positive, pleasant ones. Attention is one of the most studied cognitive processes in the social sciences. It is also one of the most difficult processes to define. There is no single definition of attention, but it is largely conceptualized as the means by which the brain chooses information (sensorial or from previously formed mental representations) for further processing (Weber et al., 2009). Attention considering brain networks’ connectivity and its variations, has been studied using cross-talk simultaneous EEG-fMRI technics (Walz et al., 2013). Psycho-neuro-physiological mechanisms associated with flow experiences have started to be elucidated (de Sampaio Barros et al., 2018; Yu et al., 2023), but its underlying neural correlates are yet to be better studied and established. Attempts to explain flow experiences in terms of brain connectivity networks have used theories such as the Multiple Demand (MD) network (Harris, Vine & Wilson, 2017b). Another theories, such as the transient hypofrontality hypothesis (Dietrich, 2003) and the synchronization theory of flow (STF) (Huskey, Wilcox & Weber, 2018; Weber et al., 2009; Weber & Fisher, 2020) were specifically developed to suggest how the brain could behave during flow. The MD theory, as well as the STF, suggests that flow results from larger activation of the focused attentional control neural network, involving mainly the dorsal attention network (DAN), with an inhibition of the salience-driven attention network, which mainly encompasses the ventral attention network (VAN), in order to prevent reorienting of attention to salient task-irrelevant stimuli (Gold & Ciorciari, 2021). As dynamic structures, brain networks present coalitions and overlapping interconnections (Pessoa, 2017, 2018), with inputs and outputs of any given region coming from, and relaying to, several cortical and subcortical areas. Furthermore, a given brain region can be affiliated to more than one network, shifting from one to another depending on task demands (Pessoa, 2018). Thus, to get a fuller understanding of neural circuit functions, it is necessary to consider both local and large-scale circuit interactions (Pessoa, 2017).

The aim of the present study was to explore changes in the psychological (qualitative attention, emotion and subjective flow experiences), peripheral-physiological (heart rate and breathing rate), and neuro-physiological (EEG analysis of power spectral density and functional connectivity), responses during a close-to-real-life of a high-risk sport situation, offering an empirical description of these intriguing experiences. Although brain functional connectivity has been more commonly investigated using fMRI approaches, we chose a portable EEG system to record neurophysiological signals, since this experiment, in a real extreme sport environment, would have been impossible to perform with fMRI. A study was conducted using EEG mobile equipment collecting brain responses during one tightrope walk at 15 m altitude (Leroy & Cheron, 2020). They used a swLORETA brain source location protocol to study brain dynamics differences between flow and stressful situations during walking. We did not perform source location since this approach relies on estimates of head shape, brain composition and different conductivity across tissues, which are difficult to obtain with high accuracy unless the corresponding MRI is available (Eom, 2023), which was not the case here. As scalp EEG analysis is simpler and its interpretation is more straight-forward, we opted to do the data analysis based on previous literature that could allow to consider the EEG electrode sites with more propensity to pick up signals on the scalp arising from specific brain networks described by pioneering fMRI technics (Luo et al., 2022; Rojas et al., 2018; Walz et al., 2013).

We chose a slackline walking protocol with varied altitudes, a high- (45 m) and a low-walk (1 m). In real life, some slackliners do the high altitude walk without safety ropes, thus configuring an imminent life-threatening situation. Our study protocol, however, did not configure a real risk situation, as participants were attached to safety ropes. Nevertheless, we tried to play-act a close to real threatening situation, exploring subjective feelings of risk according to the height of the line as a way to elicit flow experiences. The slackline, this modern form of funambulism, which has gained increased popularity over the last decade, was chosen for two reasons: first, because this activity allowed a well-controlled experimental design by manipulating the subjective feeling of risk according to the height of the line, while keeping all other parameters of performance constant; second, because participants typically move slowly, limiting the amount of motion induced-artifacts in the EEG recordings, same reason why the protocol was chosen for the tightrope study (Leroy & Cheron, 2020).

Some studies can be found in the literature addressing slackline practice, investigating the balancing capabilities of experts and beginners (Stein & Mombaur, 2022), or the metabolic demands of less and more experienced practitioners (Ueta et al., 2022). A study using a slackline walking task and subjective informed awareness, reconceptualizes flow experiences as a property of the self-perception performer-environment coupling (Montull et al., 2020). Other work, looked at functional connectivity related to slacklining (Baláš et al., 2023), but they used fMRI, which means that measurements were performed before and after the actual practice, and not during, as in the present work. Another work which has recorded brain changes during the slacklining practice used a fNIRS equipment (Seidel-Marzi et al., 2021), but they did not study the brain in highline situations.

## Materials and Methods

### Subjects

Eight adult volunteers (all males; age: 27 ± 7 years) without history of brain injury were included in the experiment. Inclusion criteria were: 1. to hold extensive slackline practice; and 2. to have already tried highline walking. All participants gave their written informed consent and were free to withdraw from the experiment at any time. The study’s protocol was approved by the scientific council of the University (CER–Comité dÉthique de la Recherche d’Université Côte d’Azur under n° 2023-002), and was performed in accordance with the ethical standards of the declaration of Helsinki.

### General procedure

The experiment was conducted outdoors in a mountainous environment next to the city of Nice, in the French Alps. This place is composed of steep rocky limestone cliffs falling straight into the Mediterranean Sea and the city of Monaco.

**Figure 1:**
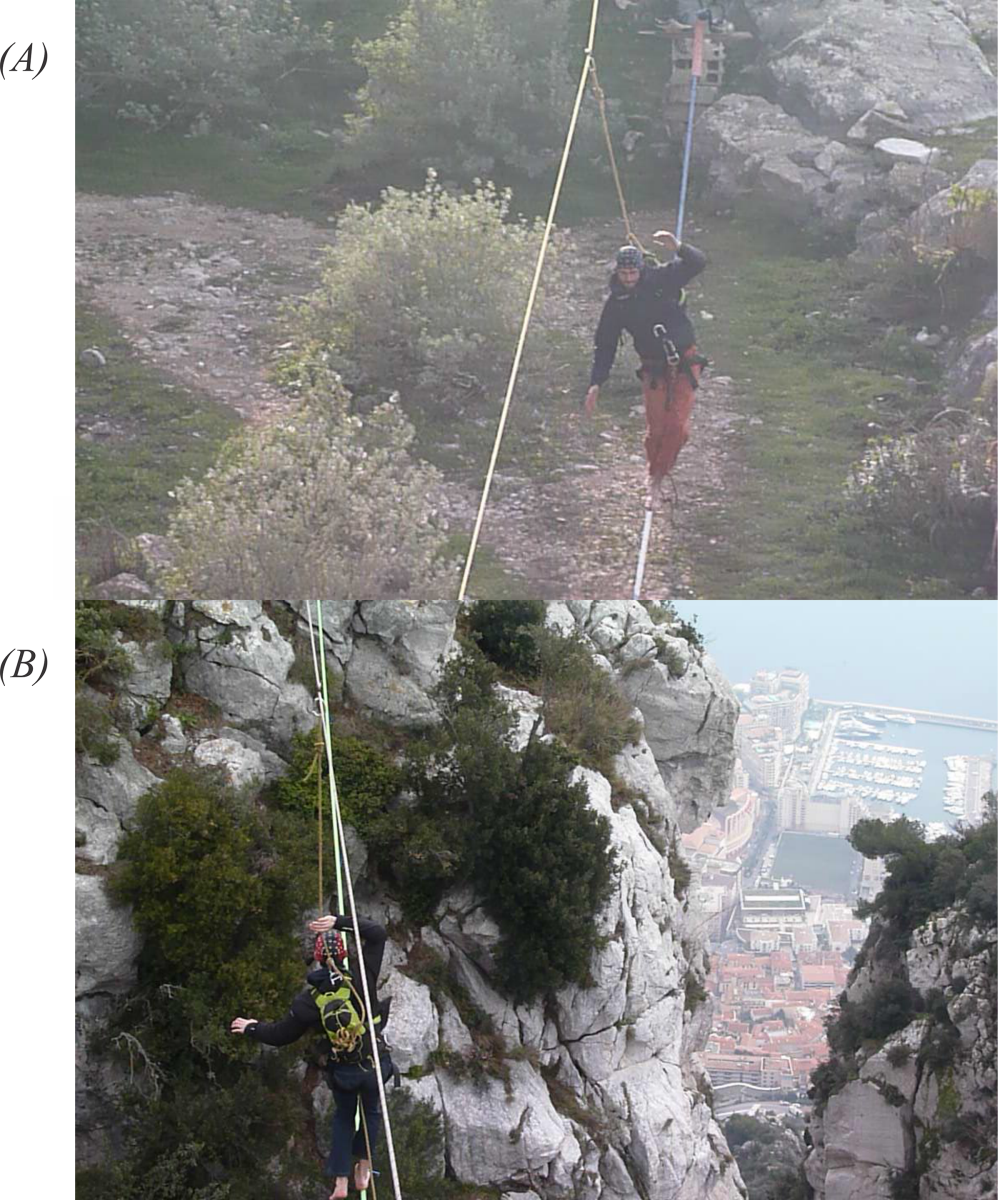
Participants walking in the slackline tapes.

Participants were first equipped with a security harness, a thoracic belt collecting cardiac and respiratory measures, the EEG headcap, and a small backpack containing the EEG data acquisition system. Then, participants were asked to complete two slackline walks on two separate lines. For each line, the given oral instructions were to traverse the line entirely and return. By essence, the implicit goal was to complete this objective while also minimizing the number of falls. The two lines were similar regarding the important parameters that influence the objective difficulty of the task. Specifically, both lines had the same 22 m length, were made from the same 5 cm width polyester sling, and were attached with the same equipment at the same tension. Above the two slacklines, a static security rope was firmly attached to prevent participants from making long falls using a safety leash connected to the back of the safety harness (Fig. 1). This safety system is somewhat different from usual practices: for the slackline at the ground, subjects normally use no security systems. In highlines, the safety rope is usually strapped just under the walk line, which leads to longer and more impressive falls as the participants fall upside down. Our different security system was chosen to limit possible damage to the equipment and to standardize conditions between the two situations. The two lines differed only in terms of the height at which they were set. One line was attached just above the ground at a height of about 1.2 m (low-walk). Note that, due to elasticity, the distance from the ground could be reduced to only 20 cm at the midpoint, when the line was loaded with the participant’s weight. The ground was flat and cleared from any obstacle. For the highline, the line was 45 m above a steep gully descending straight to the seaside, giving the visual impression of a 500 m height (high-walk) (Fig. 1B*)*. The two lines were distant by about 500 m from each other. The order of the walking condition was counterbalanced across participants. Before each walking condition, participants sat quietly for 2 min and the last 30 s counted as the preliminary rest period. Immediately after exiting each line, subjects were asked to complete a psychological questionnaire.

The whole experiment was video-recorded to mark periods of interest in the recorded biosignals (heart rate, breathing rate, EEG). During the two walking conditions, only the signals recorded when participants were able to properly stand on the line (*i.e*., without holding the leash) were retained for analysis.

### Psychological measures

#### Flow experience

The short form of the Flow State Scale 2–FSS-2 (Jackson, Martin & Eklund, 2008), rated on a 7-point Likert scale was included in the questionnaire, filled after each walking condition (Cronbach α = 0.780). The authors have permission to use this instrument from the copyright holders. This scale assesses the nine components, or key characteristics, proposed in the conceptual theory of flow (Csikszentmihalyi, 1990), which are evaluated by the answers of: “1-I felt I was competent enough to meet the high demands of the situation” (Challenge-Skill Balance); “2-I did things spontaneously and automatically without having to think” (Action-Awareness Merging); “3-I had a strong sense of what I want to do” (Clear Goals); “4-I had a good idea while I was performing about how well I was doing” (Unambiguous Feedback); “5-I was completely focused on the task at hand” (Concentration); “6-I had a feeling of total control over what I was doing” (Sense of Control); “7-The way time passed seemed to be different from normal” (Transformation of Time); “8-The experience was extremely rewarding” (Autotelic Experience); “9-I was not worried about what others may have been thinking of me or my performance” (Loss of Self-Consciousness). The answers to item 7 (Transformation of Time) led to a large decrement of the internal scale consistency, decreasing Cronbach α from 0.780 to 0.517; hence, as done in previous studies (*e.g*., Scott-Hamilton, Schutte & Brown, 2016), this item was excluded for further analysis when calculating the composite scores. The answers of the French translations to the other eight items were considered for statistical analysis in composite scores. Since recent conceptualization of flow describes it as a state of effortless attention (Bruya, 2010), two questions were added to measure the extent to which participants were able to maintain their focus of attention, and to measure the amount of subjective effort required to focus their attention (“10. I was very concentrated during the task” and “11. There was no effort to concentrate”). These items have been used in several studies (*e.g*., Csikszentmihalyi & Nakamura, 2010; Harmat et al., 2015). Items 10 (named attention) and 11 (named effortless attention), were analyzed separately from the FSS-2.

#### Positive and negative affects

A part of the psychological questionnaire assessed participants’ positive and negative aspects using the French version of the Short-Form of the Positive and Negative Affect Schedule (PANAS), (Gaudreau, Sanchez & Blondin, 2006; Thompson, 2007). This form includes two 5-item scales to measure both positive and negative affects (Cronbach α = 0.79 and 0.73 for the positive and negative aspects, respectively). Each item consists of an adjective-positive items: “Attentive, Active, Alert, Determined and Inspired”; and, negative items: “Hostile, Ashamed, Upset, Afraid and Nervous”. Using a 7-point Likert scale, participants were asked to indicate to what extent each adjective corresponded to their emotional state when they were walking on the line.

#### Physiological measures

The physiological measures (heart rate (HR) and breathing rate (BR)) were collected using the BioHarness thoracic belt (BH3; Zephyr Technology, Annapolis, USA). The electrocardiographic signal was sampled at 2 kHz through conductive silver fabric electrodes that are related to the transmitter. The HR (in beats per minute) was automatically calculated by internal algorithms of the BioHarness. The BR (in cycles per minute) was measured at 25 Hz by monitoring thoracic expansion using a strain gauge sensor. It should be noted that the reliability of the BioHarness has been adequately demonstrated for such cardiac (Kim et al., 2013) and respiratory (Hailstone & Kilding, 2011) measurements. To allow comparisons between distinct individuals, the heart and breath rates measured in each second during the tasks were normalized to their respective average during the last 30 s of rest period preceding that given task condition. Then, the rate variation for subject $i$, during task $T$ and time instant $t$ is given by Eq. (1):

(1) $${\rm \Delta}{R_{iT}}\left( t \right)\; = \; \displaystyle{{{R_{iT}}\left( t \right) - {R_{iT}}} \over {{R_{iT}}}}$$

in which ${R_{iT}}$ is the average of the rates measured at the last 30 s of the resting period.

### Electroencephalography measures

#### EEG system and data preprocessing

Cortical activity was recorded at 1 kHz using 62 electrodes (mastoid electrodes were discarded), referenced at CPz, and positioned according to the 10-10 international electroencephalography (EEG) system. We used the eego^tm^sports (ANT Neuro, Enschede, The Netherlands) system, a portable equipment with a specific construction to reduce motion artefacts, enabling brain activity recording with moving subjects. The electrode impedance was initially maintained below 5 kΩ with the use of saline gel. The equipment’s amplifier was stored in a large layer of cushioning in a small backpack.

For the first offline data analysis, the Advanced Source Analysis (ASA 4.9.3; ANT Neuro, Enschede, The Netherlands) software was used. Data were first re-referenced to the average of the 64 electrodes’ signals. Next, the raw data were downsampled to 256 Hz (since we were not interested in frequencies higher than the gamma band, and higher sampling frequencies would demand greater computational power, which in this case would be unnecessary) and bandpass-filtered between 0.5–50 Hz, with filter slope at 24 dB/oct using the Butterworth Zero phase Band-Pass filter, implemented in ASA 4.9.3. Afterwards, the data were exported to the eeg BRAIN Vision format, to be further processed using EEGLab, a MATLAB toolbox (Delorme & Makeig, 2004). The recorded time-series were visually inspected for motion artifacts, and segments which were clearly disrupted by movement (*e.g*., which contained too many high-frequency and large-amplitude oscillations) were discarded: about 10–30% of data were excluded, depending on the subject; average recording time between subjects was of (8.1 ± 1.7) min, which accounts for (124,416 ± 26,112) data points in the EEG signals. Data points prior and post any discarded segments were concatenated to proceed with the preprocessing. Following manual signal exclusion, independent component analysis (ICA) (Hyvärinen & Oja, 2000) was used to separate the signal into 62 sources. Each independent component was then visually analyzed to identify the ones that contained mainly noise, which were excluded from the signals’ reconstruction. Finally, data were band-pass filtered into four traditional frequency bands studied in the EEG literature: *θ* (4–8 Hz), α (8–13 Hz), *β* (13–30 Hz) and γ (30–50 Hz). For the electrodes’ topography, the coordinates of the Talairach Atlas Space System (Koessler et al., 2009; brmlab, 2011) were considered.

#### Estimates of the signal power—power spectral density

Since the band power of specific EEG frequencies relates to the task at hand (Siegel, Donner & Engel, 2012), one branch of our analysis consisted in studying how the power of distinct frequency bands was altered due to the high-walk and low-walk conditions.

The signals’ power was computed for each electrode, condition and frequency band using the Welch’s transform (Welch, 1975). To enable comparisons between individuals, power values during the tasks were normalized to their average during the rest period preceding that given task, in the same way as done for HR and BR. In other words, the 30 s rest immediately prior to either the high or low-walk was considered as a baseline measurement for each subject, with the following power variations being computed in relation to it for every 1s-segment the task was being performed. Mathematically, then, the power variation in electrode $e$ during task $T$, for each frequency band, subject and time instant $t$ $\left[ {{\rm \Delta}{P_{e,T}}} \right]$ can be written as:

(2) $${\rm \Delta}{P_{e,T}} = \displaystyle{{{P_{e,T}}\left( t \right) - {P_{e,T}}} \over {{P_{eT,}}}}$$

In Eq. (2), ${P_{e,T}}\;$denotes the median power at the baseline state for that specific subject, frequency band, electrode and task. Note that, when computed in this manner, ${\rm \Delta}{P_{e,T}}\left( t \right)$ actually represents relative variations to rest, with positive (negative) values corresponding to power increases (decreases).

#### Functional connectivity analysis

Functional connectivity assumes that there may be a relation between the activity of two distinct brain areas, even when there is no anatomic linkage between them (Rogers et al., 2007). A large variety of measures have been proposed to estimate FC in EEG signals (Bastos & Schoffelen, 2016). In this work, we opted for the coherence, since it is amongst the most commonly employed methodologies (Bowyer, 2016) in the field. Basically, coherence measures the synchronicity between the activity of two brain regions, mainly considering their phase difference. More specifically, even if two signals are out of phase from each other, they can still present a high coherence, should their corresponding phase difference remain approximately constant (Srinivasan et al., 2007). Amplitude is also accounted for, since two out-of-phase signals will tend to cancel out, and *vice versa* (Siegel, Donner & Engel, 2012).

Coherence matrices $A\left( t \right) = {\left\{ {{a_{ij}}\left( t \right)} \right\}_{62 \times 62}}$ can be built, with each element corresponding to the coherence value between electrodes $i$ and $j$ at time $t$. Such matrices can be estimated for each subject and frequency band, for each 1s-segment of the tasks’ performance. Seeking a reliable representation of the FC pattern for each condition, the matrices were time-averaged, yielding average connectivity matrices $\bar A = \left\{ {{{\bar a}_{ij}}} \right\}$. These matrices were further binarized according to a threshold $r$ by setting:

(3) $$b{\left( r \right)_{ij}} = \; \left\{ {\matrix{ {{1,\;\;\;} {if\; \; \; {a_{ij}}} \ge \; r} \cr {{0,\;}{\;\;otherwise}} \cr } .} \right.$$

Hence, the result is a set of binary matrices $B\left( r \right) = {\left\{ {b{{\left( r \right)}_{ij}}} \right\}_{62 \times 62}}$ that can be analyzed for each $r$ value. In such a scenario, for each subject, $r$ relates to how often a given connection was present from the available samples under analysis. In our work, we set $r = 0.70$.

Following the calculations for the adjacency matrices, we assessed the resulting functional networks’ topology through two widely employed graph metrics: the nodes’ degree and clustering coefficient.

The degree for node $i$, ${k_i}$, represents the total number of connections it makes (Fornito, Zalesky & Bullmore, 2016):

(4) $${k_i} = \; \mathop \sum \limits_{j = 1}^N {b_{ij}}.$$

Thus, a node with high degree makes a large number of connections, and *vice-versa*.

On the other hand, the clustering coefficient for the ${i^{th}}$ node ( $C{C_i}$) allows complementing this information by analyzing the connections among its nearest-neighbors. It can be mathematically expressed as (Fornito, Zalesky & Bullmore, 2016):

(5) $$C{C_i} = \; \displaystyle{{2\mathop \sum \nolimits_{j = 1}^N \mathop \sum \nolimits_{l = 1}^N {b_{ij}}{b_{jl}}{b_{il}}} \over {{k_i}\left( {{k_i} - 1} \right)}}.$$

This measure then expresses local connectivity between nearest-neighbors.

Relative metrics’ variations were investigated by analyzing how they varied in relation to their average resting values, similarly to the analysis done for the PSD based on Eq. (2).

### Statistical analysis

Data for psychological measures consisted of two answers (one for each task condition) per individual. The Wilcoxon test (Gehan, 1965), a nonparametric test for paired data, was used.

For physiological and EEG measures, the last 30 s of the rest period before the walking condition was considered as baseline for the statistical analysis. Mixed models were used to account for the non-independence of the repeated measures grouped within participants, an approach highly recommended for repeated measurements analysis (Baayen, Davidson & Bates, 2008). The mixed model included fixed factors representing the tasks (low- or high-walk), the order in which they were performed (first low- or first high-walk), and an interaction term between the tasks’ factor and the order of the tasks, when statistically significant. As random effects, we included a random intercept and a random slope for the task effect, that could vary for each participant. Fixed effects were considered significant at a global alpha level of 0.05 and Bonferroni method for multiple comparisons was applied to correct the *p*-values. The goodness of fit of the models was assessed by the residuals’ analysis of the normalized quantile residuals (Dunn & Smyth, 1996) *via* visual inspection of the quantile-quantile plot. Moreover, we considered a t-distribution for our models, which performed better than a classical normal mixed model.

All statistical analyses were performed in *R* (R Core Team, 2019), and the models were adjusted by the *R* package gamlss (Rigby & Stasinopoulos, 2005).

### Mixed models

To consider both random and fixed effects, our designed linear mixed models sought significant differences in HR, BR, PSD and FC, between the high- and low-walk conditions. This approach also enabled us to test whether the tasks performance’s order was relevant.

Considering a response variable ${Y_{ijt}}$ representing a measure (ΔHR, ΔBR, ${\rm \Delta}P$, ${\rm \Delta}{k_i}$ or ${\rm \Delta}C{C_i}$) of subject $i$ ( $i = 1,\; 2,...,8$), performing task $j$ (low- or high-walk) at time $t$ ( $t = 1,\; 2,...,{T_{ij}}$); ${T_{ij}}$ represents the total time participant $i$ took to perform task $j$. We assumed ${Y_{ijt}}$ as having a t-Student distribution, with the model given by the following equation:

(6) $${Y_{ijt}}\; = \; \left( {{b_{0i}} + {\beta _0}} \right)\; + \; {\beta _1}{X_{1i}}\; + \; \left( {{b_{2i}} + {\beta _2}} \right){X_{2i}}\; + \; {\beta _3}{X_{1i}}{X_{2i}} + {\varepsilon _{ijt}}.$$

In Eq. (6), ${X_{1i}}$ indicates the order in which subject $i$ performed the tasks, being equal to $1$, if the high-walk was performed first, and $0$, otherwise. ${X_{2i}}$ represents the task under analysis, being equal to one, if high-walk is currently under analysis, and 0, otherwise. ${\beta _0}$ is an intercept effect; ${\beta _1}$ represents the effect of tasks’ order; ${\beta _2}$ represents a task effect; ${\beta _3}$ indicates an interaction effect between the tasks performance’s order and the task being currently performed; ${b_{0i}}$ and ${b_{2i}}$ are random variables related to intra-subject variability, associated to the intercept term and to the slope of the task currently being performed, respectively. We assumed that ${b_{0i}}\sim N\left( {0,\;\sigma _0^2} \right)$ and ${b_{2i}}\sim N\left( {0,\;\sigma _2^2} \right)$, with $\sigma _0^2$ and $\sigma _2^2$ representing variances across individuals. Finally, ${\epsilon _{ijt}}$ is a random error variable, with ${\varepsilon _{ijt}}\sim N\left( {0,\;{\sigma ^2}} \right)$.

## Results

In the following, the acronyms HW and LW will be used throughout the text to indicate high-walk and low-walk conditions, respectively.

### Psychological parameters

Concerning subjective flow experiences, HW presented Δ = 4.33 (61.9% of the total possible score) on the flow FSS-2 scale. LW showed a higher score on the FSS-2, Δ = 5.56 (79.43% of the total possible score), displaying a significant effect in relation to HW (Wilcoxon test, *p* = 0.014, mean difference = 1.656). For the concentration of attention and for the lack of effort to maintain attention (effortless), the Wilcoxon test did not reveal significant condition effects (*p* = 0.072 and *p* = 0.203, respectively). With respect to the Short Form of PANAS, the mean of the positive aspects did not show significant differences between conditions (Wilcoxon test, *p* = 0.527). For the mean of the negative emotions experienced in the task, HW displayed a higher score in relation to LW (mean difference = 2.05; Wilcoxon test, *p* = 0.008).

### Physiological parameters

**Figure 2:**
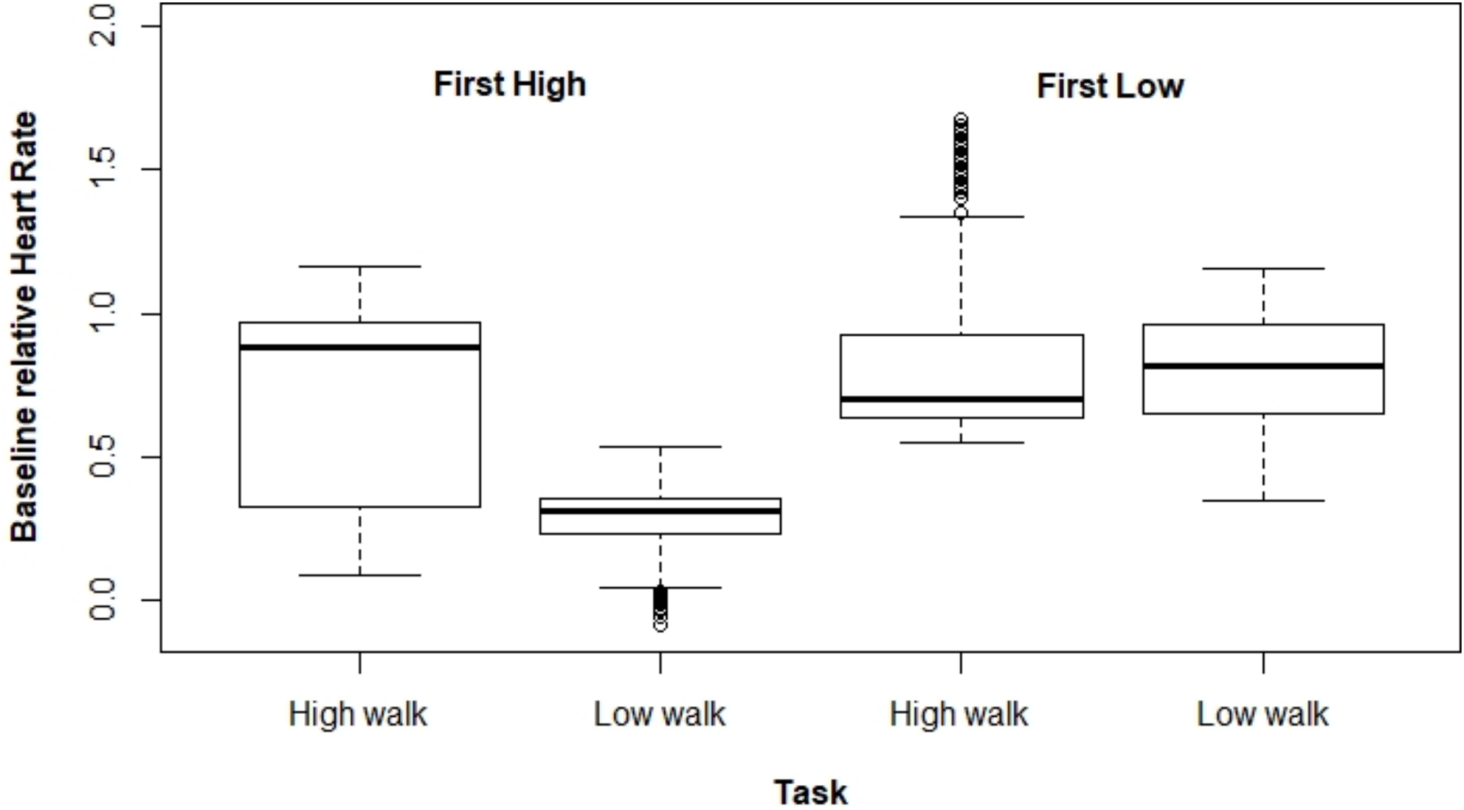
Boxplot for ΔHR according to the tasks and their order of execution.

**Figure 3:**
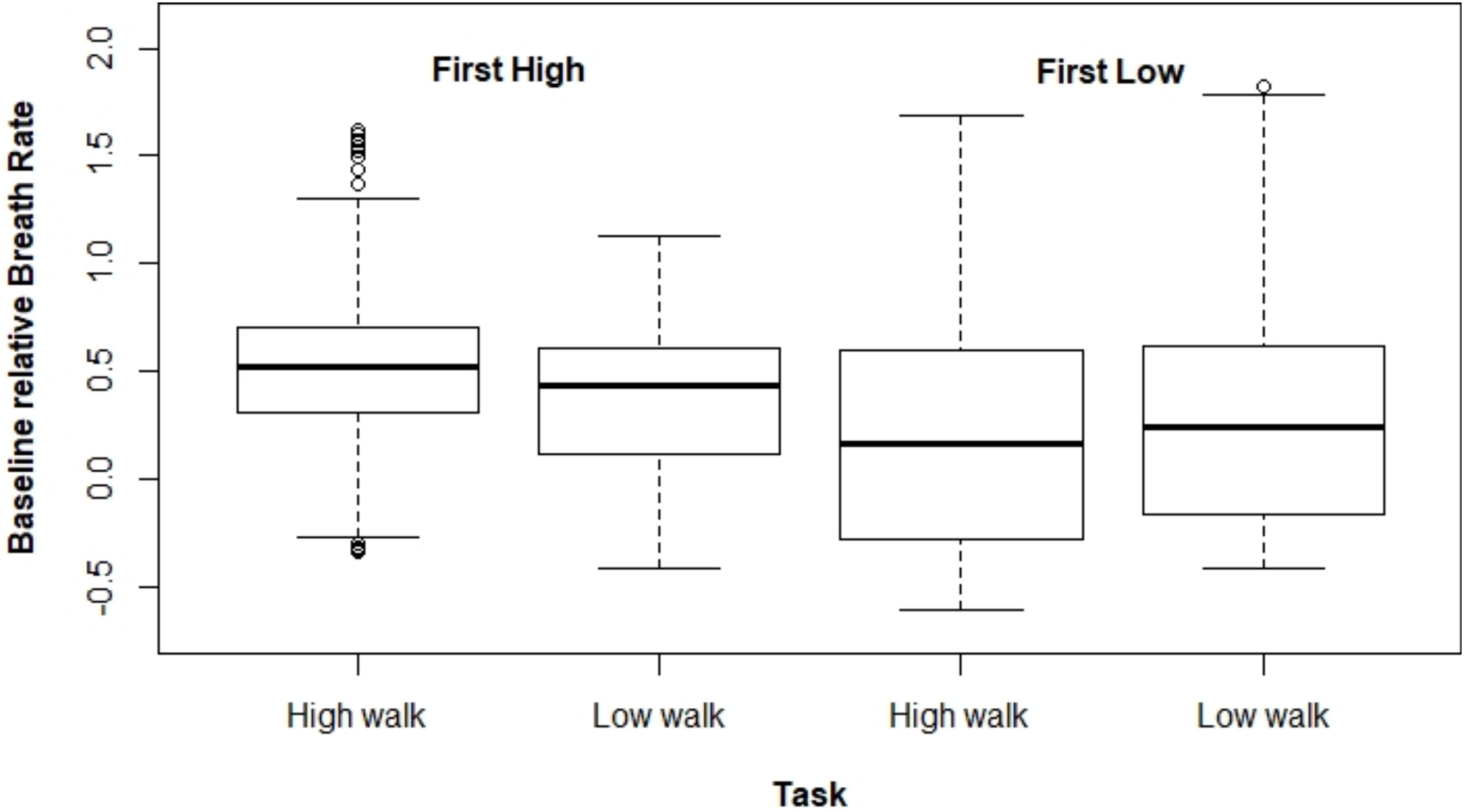
Boxplot for ΔBR according to the task and the order the tasks were performed.

Figures 2 and 3 present ΔHR and ΔBR boxplots for the measures of each subject according to the tasks and their executed order, respectively.

Regarding ΔHR, we observed a larger difference between the tasks when HW was performed first. The mixed model for ΔHR presented a significant interaction effect between the performed task and the order the tasks were executed (*p* < 0.001). The average ΔHR was higher at HW; however, the ΔHR difference between both tasks varied drastically depending on which type of walking was performed first, being 0.464 for HW executed first, and 0.045 for the other case (Table 1).

**Table 1:** Mean estimates for ΔHR according to the tasks order and the performed task obtained from the fitted mixed model.

Order	Task	Estimate	SE	CI 95%
HW first	HW	0.736	0.002	[0.731–0.741]
	LW	0.272	0.002	[0.268–0.276]
LW first	HW	0.865	0.003	[0.860–0.870]
	LW	0.820	0.003	[0.815–0.826]

**Note:**

HW, high-walk; LW, low-walk; SE, standard error; CI, confidence interval.

The mixed model adjusted for ΔBR also presented a significant interaction effect between the task performed and the tasks order (*p* < 0.001). In the same way as for ΔHR, ΔBR was higher at HW when this task was carried out first (estimated mean difference = 0.155). However, for the other scenario (*i.e*., performing LW first), there was no significant difference between both tasks (estimated mean difference = –0.044). Moreover, we observed that ΔBR was larger when HW was performed first (Table 2).

**Table 2:** Estimates of mean ΔBR according to the order and the task performed obtained from the fitted mixed model.

Order	Task	Estimate	SE	CI 95%
HW first	HW	0.522	0.010	[0.502–0.541]
	LW	0.367	0.010	[0.348–0.386]
LW first	HW	0.229	0.012	[0.205–0.252]
	LW	0.273	0.014	[0.246–0.301]

**Note:**

HW, high-walk; LW, low-walk; SE, standard error; CI, confidence interval.

### Neural activity results

#### Power spectral density

**Figure 4:**
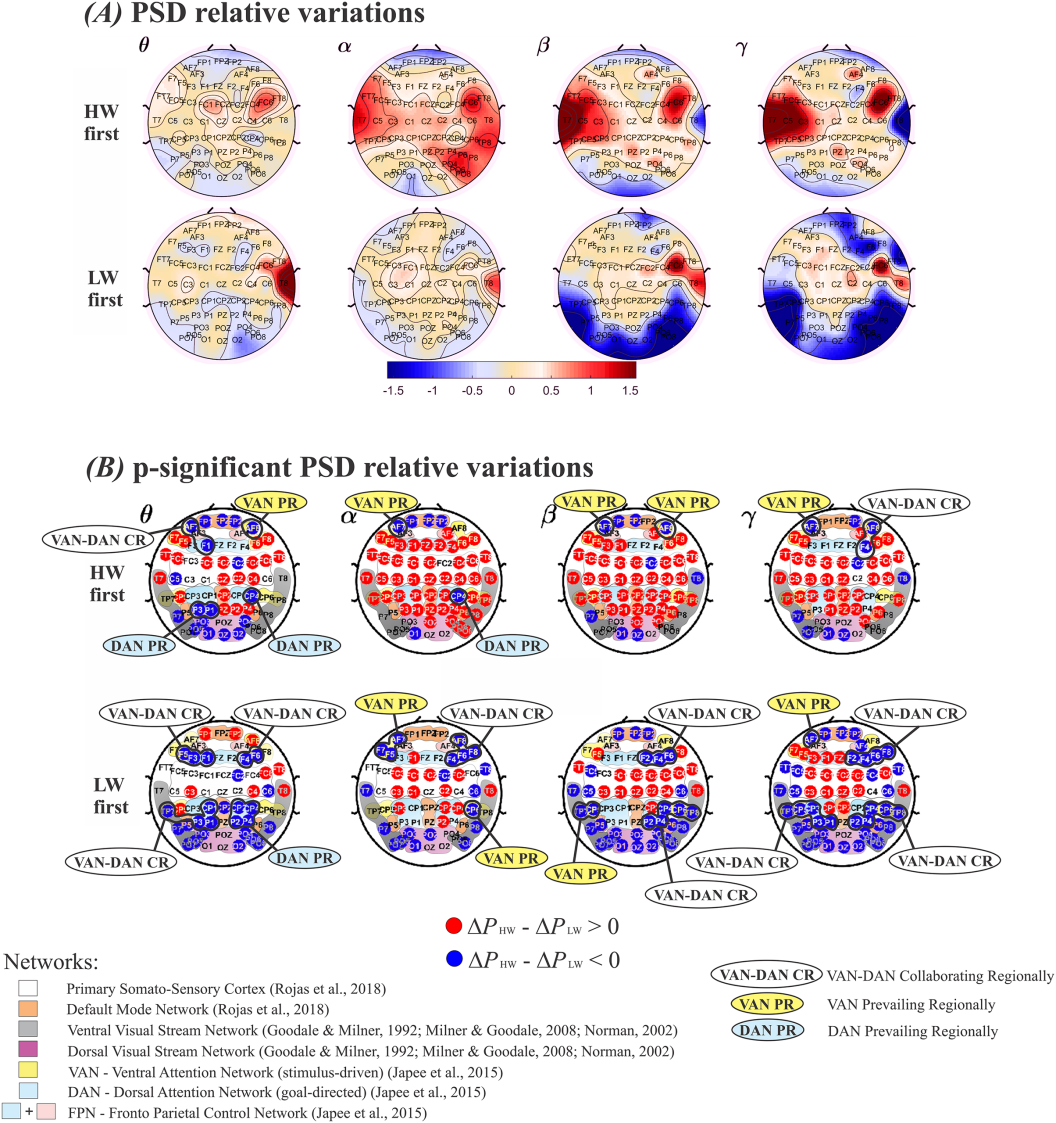
Plots of (Δ*P*_HW_–Δ*P*_LW_) according to the model in Eq. (6). (A) Positive values represent a larger relative power (Δ*P*) during HW, and *vice-versa*. Top row: HW executed first; bottom row: LW executed first. (B) Electrodes whose (Δ*P*HW–Δ*P*LW) differences were statistically significant. Top row: HW executed first; bottom row: LW executed first. The color scheme follows the setup shown in Fig. 4(A): red (blue) circles indicate HW (LW) statistically meaningful effects. The corresponding functional network more likely to encompass each electrode is also shown: Primary Somato-Sensory Cortex (white region); DMN (orange region) (Rojas et al., 2018) ; Ventral Visual Stream Network (grey regions); Dorsal Visual Stream Network (purple region) (Goodale & Milner, 1992; Norman, 2002; Milner & Goodale, 2008); VAN (yellow regions); DAN (soft blue regions); FPN (soft blue + pink regions) (Japee et al., 2015). HW, high-walk; LW, low-walk. Regarding LW, the same brain hemispheric significant Δ*P* simultaneously at frontal or parietal-temporal lobes within VAN and DAN, are marked as ‘collaborating regionally’ (CR). Electrodes related to only one attention network, presenting higher significant Δ*P* on these specific close areas, are marked as VAN or DAN ‘prevailing regionally’ (PR).

Since the order in which the tasks were performed was relevant, mean ${\rm \Delta}P$ estimates, obtained through the model in Eq. (6), were compared across the two walking situations. Subjects were divided in two groups–those who performed HW first and those who performed LW first–, which were analyzed separately. In each case, average ${\rm \Delta}P$ values were subtracted between HW and LW. These results are displayed in Fig. 4, in which plotted values correspond to ( ${\rm \Delta}{P_{{\rm HW}}} - {\rm \Delta}{P_{{\rm LW}}}$). The top row in Figs. 4A and 4B shows results for performing HW first, whereas the bottom row indicates the other situation. Frequency bands (*θ*, *α*, *β*, *γ*) are displayed in columns. Visualizing results in this manner enables enhancing differences across the walking conditions at the group level while considering the tasks’ order. Thus, in Fig. 4A, red (positive values) tones indicate ${\rm \Delta}{P_{{\rm HW}}} \;> \; {\rm \Delta}{P_{{\rm LW}}}$, and blue (negative values) tones indicate ${\rm \Delta}{P_{{\rm HW}}} \;< \; {\rm \Delta}{P_{{\rm LW}}}$. In other words, a mostly blue plot indicates predominance of larger ${\rm \Delta}{P_{{\rm LW}}}$ values, and *vice versa*.

Figure 4B displays electrodes whose ( ${\rm \Delta}{P_{{\rm HW}}} - {\rm \Delta}{P_{{\rm LW}}}$) were statistically significant. Red (blue) circles indicate significantly larger (smaller) ${\rm \Delta}{P_{{\rm HW}}}$ values. Smooth colored regions under electrodes’ locations represent brain functional networks: Primary Somato-Sensory Cortex (white region); Default Mode Network-DMN (orange region) (Rojas et al., 2018); Ventral Visual Stream Network (grey regions); Dorsal Visua. Stream Network (purple region) (Goodale & Milner, 1992; Milner & Goodale, 2008; Norman, 2002); Ventral Attention Network−VAN (yellow regions); Dorsal Attention Network-DAN (soft blue regions); and Fronto Parietal Control Network−FPN (soft blue + pink regions) (Japee et al., 2015). Regarding our higher flow scores, *i.e*., in LW, when both attention networks VAN and DAN, displayed significant $\Delta P$ simultaneously at the same brain hemisphere close areas (*i.e*., yellow and soft blue ‘sub-networks’ at frontal or parietal-temporal regions), it were marked as ‘collaborating regionally’ (CR), or, conversely, when electrodes related to only one, VAN or DAN, presented higher significant $\Delta P$, the regions were marked as ‘prevailing regionally’ (PR). It can be seen that regardless of condition order performance, when VAN-DAN CR was not the case, hemispheric VAN PR appeared at frontal regions in all bands but *α* for the right when HW was first, and *β* for the left when LW was the case, evidencing left lateralization tendency. There was no DAN PR at frontal areas during LW (Fig. 4B) top and bottom rows. Differently, the behavior of VAN and DAN PR at the parietal-temporal regions were more dependent on which condition was performed first. VAN PR was found only when LW was first. DAN PR was found in the two slower frequency bands, *θ* and *α*, tending to the right hemisphere. Considering yet, the most intense significant $\Delta P$ in the faster frequency bands, *β* and *γ* when LW was first (deeper intense blue in Fig. 4A, bottom row), there was no clear consistent VAN or DAN overall PR at parietal-temporal areas in LW.

From Fig. 4, it can be seen that some areas changed according to the condition carried out first, despite whether other brain regions remained approximately the same, wherever HW or LW presented significantly larger ${\rm \Delta}P$ effect regardless of which task was executed first. In the Supplemental Material, see Table 3, presenting the functional networks according to references (Goodale & Milner, 1992; Japee et al., 2015; Milner & Goodale, 2008; Norman, 2002; Rojas et al., 2018), and corresponding electrodes which showed significant differences among conditions.

**Table 3:** Brain networks and corresponding (electrodes) that presented significant differences between PSD variations (Δ*P*) for HW and LW, according to the condition executed first and to the frequency band.

		Effect	
Order	Freq. band	${\Delta P_{HW}-P_{LW} \; > \; 0}$	${\Delta P_{HW}-P{LW} \; < \; 0}$
HW first	*θ*	*PSSC* (FCz, FC1, FC4, FC5, FC6*, Cz, C2, C4*)	*PSSC* (FC2*, C5)
		*DMN* (CPz, Pz)	*DMN* (FPz, FP1, FP2)
		*VVSN* (T7)	*VVSN* (P7*, PO5*)
		*VAN* (F5, F6, F7, F8, CP5*)	*DVSN* (PO3*, PO4*, Oz, O1, O2*)
		*FT* (FT7, FT8*)	*VAN* (AF7, AF8)
			*DAN* (F1*, CP4*, P1*, P3*)
	α	*PSSC* (FCz*, FC1*, FC3*, FC4*, FC5, FC6, Cz, C1*, C2*, C3*, C4, C5, C6)	*DMN* (FPz, FP1, FP2)
		*DMN* (CPz, Pz, P6)	*DVSN* (O1*)
		*VVSN* (T7, T8*, TP7, TP8, P8, PO6, PO8)	*VAN* (AF7*)
		*DVSN* (POz, PO4)	*DAN* (CP4)
		*VAN* (F5, F6, F7, F8, CP5, CP6, TP7, TP8)	
		*DAN* (Fz, F1*, F2, F3, F4, CP1, CP2*, CP3*, P1, P2*, P3, P4)	
		*FPN* (same as DAN + AF4)	
		*FT* (FT7, FT8)	
	*β*	*PSSC* (FCz*, FC1*, FC3, FC4*, FC5, FC6*, Cz*, C1*, C2*, C3*, C4*, C5, C6)	*PSSC* (FC2)
		*DMN* (CPz, Pz, P6)	*DMN* (FPz*, FP1*)
		*VVSN* (T7, TP7, TP8)	*VVSN* (T8, P7*)
		*DVSN* (POz, PO4)	*DVSN* (Oz*, O1*)
		*VAN* (F5*, F6, F7, F8*, CP5, CP6, TP7, TP8)	*VAN* (AF7, AF8)
		*DAN* (F1, F3, CP1, CP2*, CP3, CP4, P1, P2, P3, P4)	
		*FPN* (same as DAN + AF4)	
		FT (FT7*, FT8*)	
	*γ*	*PSSC* (FCz*, FC1*, FC3*, FC4, FC5, FC6*, Cz*, C1*, C3*, C4, C5, C6)	*PSSC* (FC2)
		*DMN* (CPz, Pz, P6)	*DMN* (FP2*)
		*VVSN* (T7, TP7, TP8)	*VVSN* (T8, P7*, PO7*)
		*DVSN* (PO4)	*DVSN* (Oz*, O1*)
		*VAN* (F5*, F6, F7*, F8, CP5, TP7, TP8)	*VAN* (AF7, AF8)
		*DAN* (CP1*, CP2, CP3, P1, P2)	*DAN* (F4*)
		*FPN* (same as DAN + AF4)	
		*FT* (FT7, FT8)	
LW first	*θ*	*PSSC* (FC6*, C3, C4*)	*PSSC* (FC2*, C6)
		*DMN* (FP1, FP2)	*DMN* (P5)
		*VVSN* (T8)	*VVSN* (TP7, P7*, P8, PO5*, PO6, PO7, PO8)
		*VAN* (AF8, CP5*)	*DVSN* (PO3*, PO4*, O2*)
		*FT* (FT8*)	*VAN* (F5, F6, TP7)
			*DAN* (F1*, F3, F4, CPz, CP1, CP2, CP4*, P1*, P2, P3*, P4)
	α	*PSSC* (FCz*, FC1*, FC3*, FC4*, C1*, C2*, C3*)	*PSSC* (FC2, C6)
		*VVSN* (T8*)	*VVSN* (T7, P7, P8, PO5, PO7, PO8)
		*DAN* (F1, CP2*, CP3*, CP4, P2*)	*DVSN* (POz, PO3, O1*)
			*VAN* (AF7*, AF8, F5, F6, F7, F8, CP6)
			*DAN* (F4); FT (FT8)
	*β*	*PSSC* (FCz*, FC1*, FC2, FC4*, FC6*, Cz*, C1*, C2*, C3*, C4*)	*PSSC* (FC5, C5, C6)
		*VVSN* (T8)	*DMN* (FPz*, FP1*, FP2, P5, P6)
		*VAN* (F5*, F8*)	*VVSN* (T7, TP7, TP8, P7*, P8, PO5, PO6, PO7, PO8)
		*DAN* (CP2*)	*DVSN* (POz, PO3, PO4, Oz*, O1*, O2)
		*FT* (FT7*, FT8*)	*VAN* (F6, CP5, CP6, TP7, TP8)
			*DAN* (F2, F4, CP4, P2, P4)
	*γ*	*PSSC* (FCz*, FC1*, FC2, FC3*, FC6*, Cz*, C1*, C2, C3*)	*PSSC* (FC4, FC5, C5, C6)
		*VVSN* (T8)	*DMN* (FPz, FP1, FP2*, P5, P6)
		*VAN* (F5*, F7*)	*VVSN* (T7, TP7, TP8, P7*, P8, PO5, PO6, PO7*, PO8)
		*DAN* (Fz, F1, F3, CP1*)	*DVSN* (PO3, PO4, Oz*, O1*, O2)
			*VAN* (F6, F8, CP5, CP6, TP7, TP8)
			*DAN* (F2, F4*, CP3, CP4, P1, P2, P3, P4)
			*FPN* (same as DAN + AF4)
			*FT* (FT7, FT8)

**Note:**

HW, high-walk; LW, low-walk; PSSC, primary somato-sensory cortex; DMN, default mode network; VVSN, ventral visual stream network; DVSN, dorsal visual stream network; VAN, ventral attention network; DAN, dorsal attention network; FPN, fronto parietal control network; FT, fronto temporal regions. Electrodes presenting significantly larger (*ΔP*) effect in the same frequency band regardless task order are marked by *.

#### Functional connectivity

**Figure 5:**
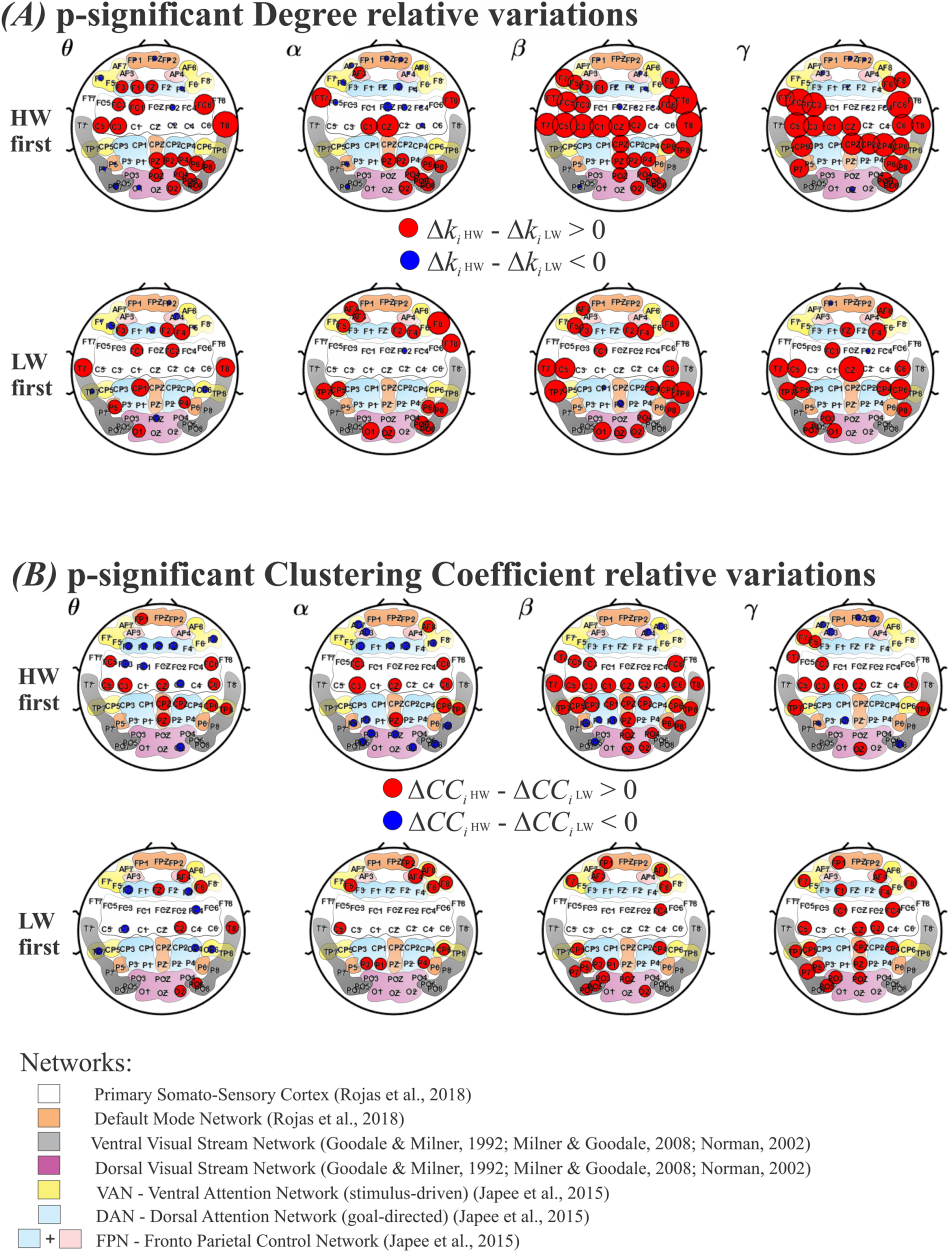
Variations for the graph metrics. (A) Electrodes whose degree variation differences (ΔK_i HW_–ΔK_i LW_) were statistically significant according to the model in Eq. (6). Top row: HW executed first; bottom row: LW executed first. (B) Electrodes whose clustering coefficient variation differences (ΔCC_i HW_–ΔCC_i LW_) were statistically significant according to the model in Eq. (6). Top row: HW executed first; bottom row: LW executed first. Red (blue) circles indicate HW (LW) statistically meaningful effects. The radius size represents the intensity of change in each metric. Corresponding functional networks are shown in the same scheme defined in Fig. 4. HW, high-walk; LW, low-walk.

Results for the degree and the clustering coefficient (CC), are shown in Figs. 5A and 5B, respectively. As in Fig. 4, the top row shows results for performing HW first, whereas the bottom row indicates LW first. Each frequency band (*θ*, *α, β, γ*) is displayed as columns. Electrodes with statistically significant ( ${\rm \Delta}{k_{i{\rm \; HW}}} - {\rm \Delta}{k_{i{\rm \; LW}}}$) or $({\rm \Delta}C{C_{i{\rm \; HW}}} - {\rm \Delta}C{C_{i{\rm \; LW}}}$) values are plotted as: red circles, for larger ${\rm \Delta}{k_{i{\rm \; HW}}}$ or ${\rm \Delta}C{C_{i{\rm \; HW}}}$; and as blue circles, for larger ${\rm \Delta}{k_{i{\rm \; LW}}}$ or ${\rm \Delta}C{C_{i{\rm \; LW}}}$. The radius’ size was normalized between HW and LW and represents the intensity of significance between the two conditions. Under the electrodes’ positions, smooth colored regions refer to the same brain functional networks as before (see Fig. 4).

Throughout frequency bands, regardless of which condition was performed first, Figs. 5A and 5B show greater significant electrode areas at most networks for HW in both FC graph metrics, Degree and CC, comparing to LW, as well as, overall larger intensity at the significant nodes (normalized red radius sizes in relation to blue ones).

Because of the normalized radius size, for a better view, in the Supplemental Material, see Table 4 for the Degree, and Table 5 for the CC, presenting the functional networks according to references (Goodale & Milner, 1992; Japee et al., 2015; Milner & Goodale, 2008; Norman, 2002; Rojas et al., 2018), and corresponding electrodes which showed significant differences among conditions.

**Table 4:** Brain networks and corresponding (electrodes) that presented significant differences between degree variations (Δ*k_i_*) for HW and LW, according to the condition executed first and to the frequency band.

		Effect	
Order	Freq. band	${\bf \Delta}{{k}_{{i\; }{\bf HW}}} - {\bf \Delta}{{k}_{{i}\; {\bf LW}}} \;{\bf > \; 0}$	${\bf \Delta}{{k}_{{i\; }{\bf HW}}} - {\bf \Delta}{{k}_{{i}\; {\bf LW}}} {\bf \;< \; 0}$
HW first	*θ*	*PSSC* (FC1*, FC3, FC6, C3, C5)	*PSSC* (FC2, C2)
		*DMN* (Pz, P6)	*DMN* (FPz, FP1, FP2, P5)
		*VVSN* (T8*, P8, PO6, PO8)	*VVSN* (P7, PO7)
		*DVSN* (POz, PO4, O2)	*DVSN* (O1)
		*DAN* (Fz, F1, F3*, P2, P4*)	*VAN* (F7*)
			*DAN* (F4)
	α	*PSSC* (Cz, C1)	*PSSC* (FCz, FC2*, FC4, FC5, C4)
		*DMN* (Pz)	*DMN* (FPz, FP2, P5)
		*VVSN* (P8*, PO6*, PO8*)	*VVSN* (PO7)
		*DVSN* (PO4, O2)	*VAN* (AF7, AF8, F5)
		*DAN* (P2)	*DAN* (Fz, F2, F4)
		*FPN* (*same as DAN*+AF3*)	*FPN* (*same as DAN*+AF4)
		*FT* (FT7, FT8*)	
	*β*	*PSSC* (FC3, FC5, FC6, Cz, C1, C2, C3, C5*)	*PSSC* (FCz, FC2, FC4)
		*DMN* (CPz, Pz)	*DMN* (FP1); VVSN (PO7)
		*VVSN* (T7*, T8, TP8*, PO6, PO8)	*DAN* (Fz, F2, F4)
		*DVSN* (POz, PO4*, O2*)	*FPN* (*same as DAN*+AF4)
		*VAN* (F5*, F7, F8*, CP6*, TP8)	
		*DAN* (F1, F3*, P2, P4)	
		*FT* (FT7, FT8)	
	*γ*	*PSSC* (FC3, FC5, FC6, Cz*, C1, C3, C5*, C6*)	*PSSC* (FC2*, FC4)
		*DMN* (CPz, P6)	*DMN* (FPz, FP1*, FP2)
		*VVSN* (T8, P7, P8*, PO6, PO8)	*DVSN* (Oz)
		*VAN* (F6, F8, CP5*, CP6*)	*DAN* (Fz)
		*DAN* (CP2, CP4*)	*FPN* (*same as DAN*+AF3)
		*FT* (FT7)	
LW first	*θ*	*PSSC* (FC1*, FC2)	*DMN* (FP2)
		*DMN* (P5); VVSN (T7, T8*)	*VVSN* (TP7)
		*DVSN* (O1)	*DVSN* (POz)
		*DAN* (F2, F3*, F4, CP1, P4*)	*VAN* (F5, F6, F7*, CP6, TP7)
			*DAN* (Fz)
			*FPN* (*same as DAN*+AF4)
	α	*DMN* (P6)	*PSSC* (FC2*)
		*VVSN* (TP7, P8*, PO6*, PO8*)	
		*DVSN* (OZ, O1)	
		*VAN* (F5, F8, CP5,TP7)	
		*DAN* (F2, F4)	
		*FPN* (*same as DAN*+AF3*)	
		*FT* (FT8*)	
	*β*	*PSSC* (FC1, C5*, C6)	*DMN* (Pz)
		*VVSN* (T7*, TP7, TP8*, P8)	*DAN* (CP1)
		*DVSN* (PO4*, Oz, O1, O2*)	
		*VAN* (AF7, F5*, F8*, CP6*, TP7, TP8)	
		*DAN* (F2, F3*, F4, CP4)	
	*γ*	*PSSC* (FC1, Cz*, C5*, C6*)	*PSSC* (FC2*)
		*VVSN* (T7, TP7, P8*, PO7)	*DMN* (FP1*)
		*DVSN* (PO3, O1)	
		*VAN* (AF8, CP5*, CP6*, TP7)	
		*DAN* (Fz, F4, CP4*)	

**Note:**

HW, high-walk; LW, low-walk; PSSC, primary somato-sensory cortex; DMN, default mode network; VVSN, ventral visual stream network; DVSN, dorsal visual stream network; VAN, ventral attention network; DAN, dorsal attention network; FPN, fronto parietal control network; FT, fronto temporal regions. Electrodes presenting significantly larger (Δ*k_i_*) effect in the same frequency band regardless task order are marked by *.

**Table 5:** Brain networks and corresponding (electrodes) that presented significant differences between clustering coefficient variations (Δ*CC_i_*) for HW and LW, according to the condition executed first and to the frequency band.

		Effect	
Order	Freq. band	${\bf \Delta}{C}{{C}_{{i\; }{\bf HW}}} - {\bf \Delta}{C}{{C}_{{i}\; {\bf LW}}} \; > \; 0$	${\bf \Delta}{C}{{C}_{{i\; }{\bf HW}}} - {\bf \Delta}{C}{{C}_{{i}\; {\bf LW}}} \; < \; 0$
HW first	*θ*	*PSSC* (FC5, FC6, Cz, C3, C5, C6)	*PSSC* (FC1, FC3, C2)
		*DMN* (FP1, CPz, Pz)	*DVSN* (O2)
		*VVSN* (TP8)	*VAN* (F8)
		*VAN* (CP6, TP8)	*DAN* (Fz, F1, F2, F3*)
		*DAN* (CP2)	
	α	*PSSC* (FC3, FC6, Cz, C3, C6)	*DMN* (P5)
		*DMN* (Pz)	*VVSN* (P8, PO6, PO8)
		*VVSN* (TP8)	*DVSN* (POz, PO3, O2)
		*VAN* (AF8*, CP6*, TP8)	*VAN* (AF7, F6)
			*DAN* (Fz, F2, F3, P3)
			*FPN* (same as DAN+AF3)
	*β*	*PSSC* (FC3, FC5, FC6, Cz, C1, C2, C3, C4, C5, C6)	*DMN* (P5)
		*DMN* (CPz, Pz, P6)	*VVSN* (PO5)
		*VVSN* (T7, T8, P7*, P8, TP8, P8)	*VAN* (AF8)
		*DVSN* (POz*, PO4, Oz, O2*)	*DAN* (P1, P3)
		*VAN* (CP5*, CP6, TP7, TP8)	*FPN* (same as DAN+AF4)
		*DAN* (CP1, CP2, CP3)	
		*FT* (FT7)	
	*γ*	*PSSC* (FC3, Cz*, C1, C3, C5*, C6)	*DMN* (FPz, FP2, P5)
		*VVSN* (TP7*, TP8)	*VVSN* (PO8)
		*VAN* (F5, F7*, TP7*, TP8)	*VAN* (AF7)
		*FT* (FT7)	*DAN* (P1)
			*FPN* (same as DAN+AF3)
LW first	*θ*	*PSSC* (C2)	*PSSC* (FC4, C3)
		*VVSN* (T8, PO6)	*VVSN* (TP7)
		*DVSN* (O2)	*VAN* (CP6, TP7)
		*VAN* (F6)	*DAN* (F3*, F4, CP4)
		*DAN* (Fz)	
		*FPN* (same as DAN+AF4)	
	α	*PSSC* (C5)	
		*DMN* (FP2)	
		*VAN* (AF8*, F5, F6, F8, CP6*)	
		*DAN* (P1, P3, P4)	
		*FPN* (same as DAN+AF4)	
	*β*	*PSSC* (FC4)	
		*DMN* (FP1, P5)	
		*VVSN* (P7*, PO5, PO7)	
		*DVSN* (POz*, PO3, O2*)	
		*VAN* (AF8, F6, F7, CP5*)	
		*DAN* (CP4, P1, P3)	
		*FPN* (same as DAN+AF3)	
	*γ*	*PSSC* (FC1, FC4, Cz*, C2, C5*)	
		*DMN* (FP1, CPz, Pz, P5)	
		*VVSN* (TP7*, P7, PO5)	
		*DVSN* (POz, PO3)	
		*VAN* (F7*, F8, CP5, TP7*)	
		*DAN* (F1, F4, CP4)	
		*FPN* (same as DAN+AF4)	

**Note:**

HW, high-walk, LW, low-walk. PSSC, primary somato-sensory cortex; DMN, default mode network; VVSN, ventral visual stream network; DVSN, dorsal visual stream network; VAN, ventral attention network; DAN, dorsal attention network; FPN, fronto parietal control network; FT, fronto temporal regions. Electrodes presenting significantly larger (Δ*CC_i_*) effect in the same frequency band regardless task order are marked by *.

It is interesting to note that LW presented yet less significant regions in faster frequencies when LW was performed first. This can be seen for Degree, where LW showed significance only on FC2 in the α band; while on PZ and CP1 in the *β* band; and on FC2 and FP1 in the *γ* band; and it is even more evident for CC, where LW did not present any significant sensor in the α, *β*, or *γ* band frequencies.

## Discussion

Psychological investigations have linked extreme sports with flow experiences (Breton, 2000; Brymer & Schweitzer, 2017; Jackson & Csikszentmihalyi, 1999; Moen et al., 2017; Partington, Partington & Olivier, 2009). Here we employed a slackline-based protocol with varying altitudes to promote a kind of ‘relaxed subjective tension’ during action, attempting to induce ‘(effortless) relaxed attention’ (Bruya, 2010) to elicit flow. We examined the psychological, peripheral-physiological and neurophysiological responses associated with qualitative attention, flow experience, and positive/negative emotions during a close-to-real-world extreme sport situation. We did not find statistically significant differences of subjective attention and effortless attention between HW and LW. Highest scores on the FSS-2 flow scale questionnaire were higher for LW, whereas the negative emotion aspects were higher at HW. It has been suggested that 60% of the total possible score on FSS-2 can be considered as indicating some degree of flow experience (Jackson & Eklund, 2004; Kawabata & Mallett, 2011). HW and LW presented 61.9% and 79.43%, respectively, of the total possible score on FSS-2. Considering that participants did not hold large experience with HW, while the protocol does not configure a real risk situation (due to participants being attached to safety ropes), the results on FSS-2 suggest that the subjects’ skills might have been balanced with the walking challenges to provide degrees of flow experiences, while the negative emotion aspects led to some inhibition of flow during HW.

These indications are reinforced by the higher ΔHR during HW, also suggesting that negative emotions, such as fear, tension, *etc*., yielded sympathetic activation, especially when HW was carried out first. However, when studying LW-first results, ΔHR differs considerably less between the two conditions. Complementarily, ΔBR results were also higher in HW when performing HW first. Fast BR is a sympathetic domain signal; therefore, these results indicate a collaboration of this domain to inhibit flow experiences. For LW-first, we observed an inverse trend, with LW showing slightly higher, although not significant, ΔBR than that shown in HW.

Overall, results for ΔHR and ΔBR, when considering LW first, could suggest an equilibrium between sympathetic and parasympathetic tones during HW. Indeed, other studies have found sympathetic-parasympathetic equilibrium related to flow experiences (de Manzano et al., 2010; Harmat et al., 2015; Peifer et al., 2014; Tozman et al., 2015; Ullén et al., 2010). Based on several psychological reports (*e.g*., a free solo climber (free of security ropes), compares flow as a hyper-focused and, at the same time, calming, experience (Sparks, 2016)), on these peripheral-physiological responses, and on some of our neurophysiological results, we suggest that scientific experiments using HW protocols, as well as other extreme sports, can potentially promote simultaneous sympathetic-parasympathetic engagement. This might induce calming sensations when dealing with fear control in order to be able to cope with the task at hand, leading to a propensity of flow experiences.

It is highly conceivable that the EEG “flow signature” will be polyrhythmic and supported by different mechanisms (Cheron, 2016). Hence, to better comprehend the complex brain functioning behavior through different emotions and feelings, a set of theories and points-of-view should be considered beyond EEG literature. In the following, we discuss some theoretical approaches and how its relate to the brain measures under investigation, *i.e*., relative differences in the signals’ power and functional connectivity metrics.

Mainly, our $\Delta P$ results lead us to consider that the brain’s signatures cannot be well generalized only across tasks and conditions, but must be understood as highly context-dependent, being altered by perception, cognition, emotion, motivation, action *etc*., (Pessoa, 2017). The fact that we are calculating $\Delta P$ as relative-to-baseline values (Eq. (2)), and that the average ${\rm \Delta}P$ values were subtracted between HW and LW, implies that the observed results reflect that the signal’s power in the mentioned areas undergoes actual variation when compared to the baseline condition (rest), and when contrasted between the two altitudes. Our main findings raised three hypotheses:

**(H1) The order of condition performed first leads to different** $\Delta {P}$ **lateralization between brain hemispheres.** The context of the conditions, such as the task’s order, influence psychological aspects, which reflect in $\Delta P$, such as changing lateralization increases between brain hemispheres. The hemispheric laterality valence hypothesis (Hellige, 1993) proposes dominance of the right hemisphere for processing negative, unpleasant emotions, and dominance of the left hemisphere for positive, pleasant ones. While our psychological negative emotion (PANAS) and peripheral physiological results presented signals of emotional arousal when HW was performed first, we had the left hemisphere with higher $\Delta P$ in a larger region across all frequency bands for HW. The $\Delta P$ results contradict the hemispheric laterality valence hypothesis when performing HW first. Relating to HW with LW first, our results could be considered as sustaining the lateralization hypothesis: HW showed right lateralized fronto-temporal higher $\Delta P$ through all frequency bands (Figs. 4A and 4B, bottom row), while it displayed a smaller area at the left hemisphere and central brain regions, compared to when starting with HW (Fig. 4B). But, interestingly, the peripheral physiology revealed a sympathetic/parasympathetic equilibrium in HW when LW was first, and HW presented significant $\Delta P$ at the vmPFC region, for both hemispheres, in the *θ* frequency (Fig. 4B bottom row, first column). vmPFC is related to counterbalancing amygdala activation during emotional arousal as in fear control (Pessoa, 2017, 2018). These findings could be interpreted as a reflex of a mental strategy to handle negative emotions and promote calm conduction (*i.e*., significantly higher $\Delta P$ at vmPFC only in the “slow-frequency” *θ* band and sympathetic-parasympathetic equilibrium). Concerning the hemispheric valence hypothesis and these contrary results, it could be argued that HW could also promote positive/pleasant emotions, competing with the negative. Managing and overcoming fear and anxiety can provide a medium to facilitate profound and positive transformations (Holmbom, Brymer & Schweitzer, 2017). We did not find significant results with the positive emotions on the PANAS questionnaire for any condition, but LW was related to the experience of high flow, and flow is commonly associated with enjoyment emotions (Pels, Kleinert & Mennigen, 2018; Rufi et al., 2016). Our results could suggest a LW trend towards positive emotions linked to a slightly higher $\Delta P$ at occipital, parietal and temporal left hemisphere (Figs. 4A and 4B). However, LW also showed higher $\Delta P$ in the right hemisphere, markedly at the temporal lobe in the *β* and *γ* bands when HW was first. Some inconsistencies of our results can be derived from the tasks’ performance order, which apparently influenced the involved emotions and results. Differences in $\Delta P$ between tasks were thought to be mainly due to a distinct visual stimulus of height. Previous EEG studies have reported habituation of visual responses at different stimulus conditions (*e.g*., Brust-Carmona et al., 2014; Cao et al., 2020). This might indicate a type of training saturation mechanism in the brain for similar types of tasks conducted in a relatively close time interval. It could mean that performing either of the walking conditions first causes the $\Delta P$ response at the subsequent task to be damped. It can be perceived that larger brain areas are dominated by the performed first condition (Figs. 4A and 4B). Hence, at this point, our results support that the tasks’ order cannot be disregarded in this type of study and, thus, similarly-designed experiments ought to consider this and other sorts of complexities in their hypotheses.

**(H2) Regardless of which condition is performed first, negative emotion aspects induce an increase in** $\Delta {P}$ **at the sensorimotor cortex and parietal medial areas during HW, while high scores on the flow questionnaire coincide with persistent higher** $\Delta {P}$ **at medial prefrontal, middle-frontal and occipital areas in LW.** HW, related to greater negative emotion aspects, presented higher $\Delta P$ at central brain areas, while larger flow experiences in LW correspond mainly to higher $\Delta P$ at medial prefrontal, middle-frontal and occipital cortex of both brain hemispheres. Regardless of the condition performed first, higher $\Delta P$ across frequency bands showed some characteristics that remained approximately stable for both task conditions. During HW, higher $\Delta P$ was observed at central brain areas, including the Primary Somato-Sensory Cortex (PSSC–white region in Fig. 4B), the premotor area, the medial parietal region related to the DMN (Rojas et al., 2018) (orange regions in Fig. 4B), and, mainly when starting by HW, areas of the frontoparietal control attention network (FPN) (Japee et al., 2015). The DMN is usually active during worries about the self. The higher $\Delta P$ at these regions could indicate a DMN and FPN co-activation. Worries due to an unconscious life-threatening situation, induced by the high-altitude, should promote an effortful focused attention on the “motor engram (memory trace)” for the whole-body motion (sensorimotor areas) (Ueta et al., 2022), to avoid falling during HW. LW showed higher $\Delta P$ on prefrontal sensors, for almost all frequency bands and situations (except when LW was first for the *θ* and *α* bands, Figs. 4A and 4B). Despite vmPFC being often associated to the DMN (Corbetta & Shulman, 2002; Hänsel & von Känel, 2008; Rojas et al., 2018), and commonly related to stress-relieving by counterbalancing amygdala activation (Hänsel & von Känel, 2008), the link between vmPFC and positive affective processing of safety signals has also already been shown (Harrison et al., 2017). Contrasting with the pioneer study considering EEG dynamics of flow during a tightrope performance (Leroy & Cheron, 2020), our findings do not corroborate the transient hypofrontality hypothesis (Dietrich, 2003) related with flow experiences. Other noteworthy finding related to greater $\Delta P$ during LW was observed on electrodes positioned at the middle-frontal gyrus, at both hemispheres (Fig. 4B). This gyrus has been mainly linked to the stimulus-driven ventral attention network (VAN) (Corbetta & Shulman, 2002; Harris, Vine & Wilson, 2017b). Furthermore, higher $\Delta P$ during LW was also found for the occipital area, which is related to the brain’s visual processing, as well as the starting area for visual streams pathways (Goodale & Milner, 1992; Norman, 2002; Milner & Goodale, 2008). Studies about cortical visual pathways suggest two streams (Goodale & Milner, 1992; Norman, 2002; Milner & Goodale, 2008): the ventral visual stream, directed to the temporal lobe (grey regions in Fig. 4B), and the dorsal visual stream, leading to the parietal lobe (purple region in Fig. 4B). The first is considered to be involved with objects’ visual identification and recognition, having strong connections with the limbic system which controls emotions. LW showed some tendency for higher $\Delta P$ at the left on this stream. This tendency could indicate that flow experiences do not have lateralized right brain dominance, as reported to occur in effortful attention situations (Coull, 1998; Lim et al., 2010; Nyberg & Cabeza, 2000; Ogg et al., 2008; Paus et al., 1997), running discrete and distinct neural dynamics from task engagement situations, involving both hemispheres with a slight left dominance. The other visual stream, leading to the parietal lobe, is considered involved in processing spatial location with typically ‘low self-consciousness’. The dorsal visual stream reaches the somatosensory association cortex, which interprets tactile sensory data being involved with the perception of limbs location in space, and the intraparietal sulcus, both areas considered to participate in the parietal brain region of DAN (Japee et al., 2015; Lim et al., 2010; Ogg et al., 2008) (parietal soft blue region in Fig. 4B). But, this stream also reaches the VAN at the inferior parietal lobe, more specifically at the supramarginal gyrus (CP5, CP6 electrodes), related to sensory information interpretation as well (Carlson, 2012) (posterior yellow cortex region in Fig. 4B). The VAN, also known as stimulus-driven network, can be responsible for a more opened attention to the surrounding details during a task, contributing to consider the moment-to-moment changes during action. Our current findings support an involvement of both dorsal and ventral attention networks relating to highest flow experiences.

**(H3) Flow experiences can arise as a cooperation of goal-directed and stimulus-driven brain attentional networks, with some** $\Delta {P}$ **prevalence for stimulus-driven mainly at frontal lobe.** VAN is mainly related to the middle frontal gyrus (MFG), inferior frontal sulcus (IFS) towards the inferior frontal gyrus (IFG), and to the temporo-parietal junction (TPJ) (Japee et al., 2015). DAN comprehends mainly the frontal eye fields (FEF), superior parietal lobule (SPL), and intraparietal sulcus (IPS) (Japee et al., 2015). Attempting to explain flow experiences in relation to brain networks activity, some studies consider a theory of the Multiple Demand (MD) network (Harris, Vine & Wilson, 2017b). According to this theory, the MD network overlaps VAN (*e.g*., inferior frontal sulcus) and DAN (*e.g*., intra parietal sulcus) (Duncan, 2013). As well as in STF (Huskey, Wilcox & Weber, 2018), the studies considering the MD network, further dividing attention brain networks into “sub-networks”, suggest more “activation” of brain areas associated to the goal-directed DAN, with the ‘optimal attentional control during flow’ being dependent upon the absence of disruption and monitoring processes coming from the stimulus-driven VAN (Harris, Vine & Wilson, 2017b). To verify this idea, we considered our results of high $\Delta P$ within the attention networks VAN and DAN, also divided into “sub-networks” at frontal or parietal-temporal regions, as ‘collaborating regionally’ (CR) or ‘prevailing regionally’ (PR) (Fig. 4B*)*. We further analyzed these variations concerning the relationship of the MD network attention hypothesis and our higher flow scores, *i.e*., in LW. Nevertheless, note that our $\Delta P$ analysis approach could not determine if the activity of these two networks occurred in correlated synchronization or if it occurred in an oscillatory anti-correlated way (or, yet, in a desynchronization manner), but, since slackline can be pondered as a moment-to-moment task requiring constant attention resources to body balance, we presumed it pertinent and worthwhile to discuss the above assumptions. Thus, following this proposed approach, our results (mainly at frontal lobe) suggest that, besides VAN and DAN are collaborating to flow experiences, relevantly, rather than more attached to DAN, flow experiences would tend to have more significant prevailing of VAN (Fig. 4B) top and bottom rows. Although we did not perform eye tracking measurements, characteristically the slackliners tend to maintain a ‘quiet eye’ strategy directed at a horizontal point when walking the line. Some studies have associated “quiet eye” strategies with flow experiences (Harris, Vine & Wilson, 2017a). The frontal eye field (FEF) region is important in visual attention control and eyes’ movement for seeking relevant targets or stimuli in the focus searching process (Schall, 2004). Perhaps, quiet eye strategies may possess a key role in DAN deactivation at the FEF area, and this decrease in FEF activation could allow the VAN system, mainly MFG/IFG region, to operate considering more incoming perceptions’ details with no worries to find a focus of attention. Attention would then be calmly expanded to a higher awareness of the subject’s surroundings, of the environment changing during the action, promoting the self-perception-performer-environment coupling (Montull et al., 2020).

VAN prevailing could solve the apparent paradoxical flow experiences descriptions which are difficult to conciliate when considering DAN predominance (Harris et al., 2016). Several flow reports emphasize ‘enhanced feelings and perceptions’ accompanying ‘reduced attention effort’ (effortless), contrasting at the same time with ‘lack of distraction’ and ‘superior focus’ on the task being performed. Recurring reports point to enhanced feelings and perception considerations during flow, even when apparently non-relevant to the task. A big-waves surfer describes improved perceptions of his surroundings, such as the wind, the details and variations in the face of the wave, and the salt in his mouth (Partington, Partington & Olivier, 2009). A vertical skateboarding rider mentions the feeling of being in total control, of every little movement, and very aware of everything, such as the rings in her hands (Hunter & Csikszentmihalyi, 2000). A free solo climber (climbing without safety ropes) discourses about how everything becomes elevated, with the awareness of the rock’s texture, the surrounding beauty and birds whistling (Sparks, 2016). It seems more suitable to refer to enhanced consideration of perceptions, than to enhanced perceptions themselves, as these apparently non-task relevant perceptions were present the whole time-‘salt in his mouth’, ‘rings in her hands’, ‘birds whistling’-, with subjects remaining mostly unaware of them for the most part from normal sensory feedback during tasks. In this context, when in flow experiences, an increased amount of perceptions seems to ascend to consciousness, perhaps, turning the brain stimulus-driven network (VAN) very active, tending to improve the supply information for the brain sensory association area (DAN). With the leading role of the stimulus-driven VAN, the ‘superior attentional focus’ during flow, rather than commonly understood as an acute focused attention (Csikszentmihalyi, 1990; Gold & Ciorciari, 2021), can be understood as an opened attention to more perceptions coming from the surroundings. In this way, the subject’s ‘distractions disappear’, because this superior, or, opened focus, is now handling an augmented quantity of inputs and stimuli constantly changing.

During flow, a person is often more open, alert, and flexible within the structure of the activity being performed (Harris, Vine & Wilson, 2017b). A heightened perception of the changings here and now tends to push to the present moment in a dynamical adaptive action and can be one of the catalyst that creates the opportunity to experience a flow state (Csikszentmihalyi, 1978). Action moments during flow are reported requiring to process several pieces of information in the environment simultaneously (Wiersma, 2014). Base jumpers describe flow as enabling to process more detailed sensory information. “Focus is opened in new or transcendent ways.” “[…] you’ve got a kind of sensory overload […]” “I am fully aware of everything else that is going around me. It’s the opposite of tunnel vision. […]” “You’re at this level of alertness that you’re not in a normal life.” “[…] is a feeling of existential readiness.” (Brymer & Schweitzer, 2017, p. 70).

Therefore, with the stimulus-driven network modulating the goal-directed decisions (Eckert et al., 2009), the ‘optimal attention control during flow’ would arise through the constant awakening to the dynamic adaptation opportunities. If so, flow would be denoted by a smooth continuous harmonically tuning and accurate optimal performance, overcoming habitual actions and embracing a more holistic experience.

Considering EEG graph analyses, the degree represents the amount of synchronization between a node (region) and other nodes (brain regions). The clustering coefficient reflects a similar notion, but more locally, in the sense that it evaluates the connections among a node’s neighbors. These functional connectivity variations were presented on the significant electrode sites, through the size of the circles in Fig. 5. Due to the great relative metrics in HW, for normalized results, the lesser significant electrodes in LW appeared as small representative blue circles in proportion to the HW ones (Figs. 5A and 5B). Overall, for degree and CC, LW presented lower functional brain connectivity metrics values, and covered less regions with significant electrode sites compared to HW. In contrast to the STF, which conceptualizes flow as a cognitive network synchronization process optimizing brain functioning to handle a supposed ‘limited capacity of attention’, the desynchronization among ensembles of neural populations in our functional brain connectivity investigation can be interpreted as indicating that a de-coupling of brain regions better represents flow brain signatures, while overall synchronization of brain networks indicates higher negative emotion aspects with an inhibition of flow, rather than its presence in HW. This could reflect a relaxation of constraints in global brain activity that corresponds with an expansion of the brain’s possible repertoire (thereby increasing dynamic diversity) (Kotler et al., 2022). In our case, functional connectivity was estimated through coherence analysis. Other types of potential flow-inducing experiences, such as meditation, have also been studied using the coherence metric. A study with five different meditation traditions found lower significant coherence during meditation through all groups and all studied frequency bands (Lehmann et al., 2012). The study presumed that neither increase nor decrease of EEG coherence is a virtue in itself, given that general high coherence is observed during epileptic seizures while decreased coherence was found during schizophrenic symptomatology. Interestingly, availability of dopamine D2-receptors in the striatum were positively associated with schizophrenia (Madras, 2013), as well as with flow proneness (de Manzano et al., 2013). Anyway, since coherence assesses the cooperation between system units, lower coherence implies higher functional independence of the system units (Lehmann et al., 2012). This behavior, with functional brain regions independence, may reinforce the idea of an ‘expanded model of attention’ linked to flow, when compared to the normal everyday self-imposed limited attention. The EEG recordings disclosing the brain lighting up like a “fireworks display” can suggest more perception considerations coming from VAN, timely stimulating different brain areas by the moment-to-moment changes taking place on the overabundance of information around, contributing to the time perception distortion during flow (Kotler et al., 2022). A base jumper reports that “it is a complete sensation. It’s a sensation that’s taking in all your senses and then some that you didn’t know you had” (Brymer & Schweitzer, 2017, p.68). She described it experiencing time “slowing down” with an increased ability to notice details in the environment, even while traveling at 125 miles (200 km) per hour:

[…] you experience something interesting in that your awareness of 1 s expands enormously. What we would normally perceive in 1 s is very little compared to what you perceive in 1 s on a BASE jump. Your mind, so you can deal with everything that you have to, slows things down so when you’re doing it, it feels like it’s in slow motion. […] When you watch it back on footage you look and go WOW, that’s over in a blip (clicks fingers), but when you’re doing it you know you can see the tiny little creases in the rock and different colors in the sky and you’re totally aware of where your body is in space and how its moving and … it’s very surreal. (Brymer & Schweitzer, 2017, p. 69).

Theoretically, this brain pattern could cause the ‘sense of readiness’ to adapt and act by the sudden changes happening around, creating the gap for the ‘action awareness merging feeling’, the most characteristic feeling during flow. The merging feeling then, must generate the sensation of belonging to something greater, feature found in both meditative practices (Lehmann et al., 2012) and flow experiences (Gold & Ciorciari, 2021, 2020; Sparks, 2016), reported as bliss, transcending, expanded consciousness, oceanic feelings, all-oneness, being changing together with the whole surroundings in a formlessness transient universe (Huxley, 1956).

This systemic fusion, with the self, expanded and integrated in the environment, becoming part of the same system, seems to fit well and characterizes a good consideration for the word/term labeled “‘Flow’” by the theory pioneer (Csikszentmihalyi, 1975). One of flow’s most interesting phenomenological attributes is the sensation of flow itself, often described as effortless effort, where the experience is that every action and every decision being performed leads seamlessly, perfectly, fluidly to the next (Csikszentmihalyi, 1990; Kotler et al., 2022).

## Conclusion

Some proposed neurocognitive theories of flow experiences conjecture that there is a limited-capacity of available attentional resources and suggest that flow should result from the synchronization of the focused attentional control neural network, involving mainly the DAN. To support the role of increased modulation from DAN, these theories posit an inhibition of the salience-driven attention network that cover the VAN. Considering brain behavior linked to higher flow psychological subjective results, we found VAN prevailing regionally at the frontal lobe as well as overall cortex de-synchronization. Our interpretation of these results suggests flow experiences as an opened attention to more changing details in the surroundings, *i.e*., configured as a ‘task-constantly-opened-to-subtle-information experience’, rather than a ‘task-focused experience’.

This functional state of mind would enable a deep understanding of the self as part of a larger whole, promoting the ‘action awareness merging feeling’, raising a shared identity with all universe conjoint motion, in the instance which the ‘individual member’ shares dynamic and harmonic action with everything around, improving harmonic performance as consequence. These highly synergistic dynamic experiences, fashioning jointly with the environment, match well with extreme sports in which athletes often describe the action as a partnership dance, expressing it as feeling part of nature, with enhanced senses and altered perceptions of time and space, including new ways of perceiving the body-space continuum, as if every cell was twitching and alive (Brymer & Schweitzer, 2017). These glimpse regards can raise an expanded and enlightening consideration for the concept that everything is relative. Everything is relative only because everything is the same thing: ‘movement’. By ‘movement’, everything influences everything else, while being influenced by all everything. The ‘action awareness merging flow experience insight’, or, the sensation of belonging, sharing oneness feeling with the ‘whole movement’, which actually permeates the ‘entire universe’, provide peace, calm and stillness during motion.

As practical implications, the findings of the present study provide a more comprehensive explanation of human perception during flow in extreme sports performance and suggest important insights regarding the possibility of temporal processing and spatial attention. Despite the difficulties in using sensitive equipment outside of the laboratory as well as the logistical problems, our approach attempted to emulate extreme sports’ real-world conditions. As equipment development becomes more robust to noise or waterproof, it will encourage more attempts to use extreme sports such as high-altitude slacklining, free solo climbing, wingsuits proximity flying, or giant waves riding, to study physiological and neuro-cognitive correlates of more complex and intense real experiences of flow. Improvements in equipment, allowing enhanced patterns seeking while studying flow experiences in extreme sports, could have interesting practical applications not only for extreme sports. One idea could be to develop trainings focused on promoting flow while tracking brain oscillations in traditional sports or in elite military troop missions. In these kinds of scenarios, turning on the ‘sense of readiness’ by adapting action according to the sudden changes happening around, may promote the ‘action awareness fusion’. This feeling comprises influence waves emerging from the self toward surrounding events, while perceiving actions happening in slow motion, which might, *per se*, abruptly increase performance.

### Limitations

Approaches such as ours to study brain networks inherently oversimplify the true brain network topology, where multiple regions should be functionally connected (Huskey, Wilcox & Weber, 2018), or where an ‘over request’ of one or more brain network regions influence the others; hence results should be considered with some caution. For example, our study did not involve sub-cortical interactions and it also over-simplified analyses considering the cortical areas underneath positioned electrodes. Nevertheless, we found a general brain-cortex desynchronization relative to subjective flow experience. The choice of functional connectivity measure, namely, coherence, was due to the wide use of this measure in the EEG literature; this measure has thus been extensively investigated and validated in several studies (Srinivasan et al., 2007). So, we took the opportunity to make relations with invaluable works that used the coherence approach, (*e.g*., Lehmann et al., 2012), what led us to rise interesting considerations comparing our results with other important studies (de Manzano et al., 2013; Madras, 2013). Although it may be argued that coherence suffers from volume conduction artifacts, the use of CAR (common average referencing) (Ludwig et al., 2009) in the preprocessing stage should decrease this possibility (Brunner et al., 2016; Yuksel & Olmez, 2016). The use of eye-tracking equipment could have further clarified our results, especially $\Delta P$ results at FEF area in the frontal cortex, but unfortunately we did not have access to this type of equipment at the time of data collection. The results dependence on the conditions’ order was certainly influenced by the lack of training-adaptation periods before the measurements (data collect): starting in LW might have served as a psychological preparation for the task on HW, and *vice-versa*. Nevertheless, the lack of such training-adaptation sessions was necessary in order to include as many participants as possible; in our case, eight (four on Saturday and four on Sunday). In this respect, several factors influenced on or reduced our sample size. For example, travelling to the measurement site, preparing participants with the equipment and the tasks performance itself demanded extensive time. Moreover, obtaining the appropriate authorization to use the equipment outside the laboratory environment was complicated, so that the authorization was granted only for gathering data over 1 weekend. On the other hand, this approach brought our investigation closer to real-world scenarios. In addition, all our analyses relied on powerful statistical modelling and/or seeking common trends for all subjects. Therefore, whereas the small number of subjects might preclude generalization of our conclusions, allied to the fact that this is an exploratory study which needs future works to better affirm the strength of our raised hypotheses, we are providing, to the best of our knowledge, besides the initial work evaluating one tightrope highline walking (Leroy & Cheron, 2020), an early support for seeking the corresponding psycho-physio-neurological correlates of brain behavior close to the real life in extreme sports, as well as an innovative description for this type of data, something that can certainly be replicated, improved and augmented in future studies on this research area.

## Supplemental Information

10.7717/peerj.17743/supp-1 Supplemental Information 1 Highline walking example.

10.7717/peerj.17743/supp-2 Supplemental Information 2 Lowline walking example.

## Additional Information and Declarations

### Field Study Permissions

The following information was supplied relating to field study approvals (*i.e*., approving body and any reference numbers): The study’s protocol was approved by the scientific council of the University (CER–Comité dÉthique de la Recherche d’Université Côte d’Azur), under the supervision of professor Rémi Radel, Lecturer at the University of Nice Sophia Antipolis Côte D’azur, and under responsibility of professor Raphael Zory, director of Laboratoire Motricité Humaine Expertise Sport Santé-LAMHESS

### Data Availability

The following information was supplied regarding data availability: The data is available at Zenodo: de Sampaio Barros, M. F., Stefano Filho, C. A., Toffoli de Menezes, L., Araújo-Moreira, F. M., Trevelin, L. C., Pimentel Maia, R., Radel, R., & Castellano, G. (2023). Psycho-physio-neurological correlates of qualitative attention, emotion and flow experiences in a close-to-real-life extreme sports situation: low- and high-altitude slackline walking [Data set]. Zenodo. https://doi.org/10.5281/zenodo.8172597.

## References

[ref-1] Allison AL, Peres JC, Boettger C, Leonbacher U, Hastings PD, Shirtcliff EA (2012). Fight, flight, or fall: autonomic nervous system reactivity during skydiving. Personality and Individual Differences.

[ref-2] Baayen RH, Davidson DJ, Bates DM (2008). Mixed-effects modeling with crossed random effects for subjects and items. Journal of Memory and Language.

[ref-3] Baláš J, Klaus J, Gajdošík J, Draper N (2023). Metabolic demands of slacklining in less and more advanced slackliners. European Journal of Sport Science.

[ref-4] Bastos AM, Schoffelen JM (2016). A tutorial review of functional connectivity analysis methods and their interpretational pitfalls. Frontiers in Systems Neuroscience.

[ref-5] Bowyer SM (2016). Coherence a measure of the brain networks: past and present. Neuropsychiatric Electrophysiology.

[ref-6] Breton D (2000). Playing symbolically with death in extreme sports. Body & Society.

[ref-7] brmlab (2011). Closest 10-10 Electrode position to each Brodmann area. https://brmlab.cz/project/brain_hacking/broadmannarea.

[ref-8] Brunner C, Billinger M, Seeber M, Mullen TR, Makeig S, Lansky P, Cohen MX, Özkurt TE (2016). Volume conduction influences scalp-based connectivity estimates. Frontiers in Computational Neuroscience.

[ref-9] Brust-Carmona H, Valadez G, Galicia M, Flores-Ávalos B, Sánchez A, Espinosa R, Yáñez Ó (2014). Desynchronization/synchronization of parasagittal EEG rhythms during habituation to photostimulation in adults. Revista de Investigacion Clinica.

[ref-10] Bruya B (2010). Effortless attention: a new perspective in the cognitive science of attention and action. Effortless Attention.

[ref-11] Brymer E, Feletti F, Monasterio E, Schweitzer R (2020). Editorial: understanding extreme sports: a psychological perspective. Frontiers in Psychology.

[ref-12] Brymer E, Schweitzer RD (2017). Evoking the ineffable: the phenomenology of extreme sports. Psychology of Consciousness: Theory, Research, and Practice.

[ref-13] Cao Z, Ding W, Wang YK, Hussain FK, Al-Jumaily A, Lin CT (2020). Effects of repetitive SSVEPs on EEG complexity using multiscale inherent fuzzy entropy. Neurocomputing.

[ref-14] Carlson NR (2012). Physiology of behavior.

[ref-15] Chatterton RT, Vogelsong KM, Lu YC, Hudgens GA (1997). Hormonal responses to psychological stress in men preparing for skydiving. Journal of Clinical Endocrinology and Metabolism.

[ref-16] Cheron G (2016). How to measure the psychological flow? A neuroscience perspective. Frontiers in Psychology.

[ref-17] Clough P, Houge Mackenzie S, Mallabon L, Brymer E (2016). Adventurous physical activity environments: a mainstream intervention for mental health. Sports Medicine.

[ref-18] Corbetta M, Shulman GL (2002). Control of goal-directed and stimulus-driven attention in the brain. Nature Reviews Neuroscience.

[ref-19] Coull JT (1998). Neural correlates of attention and arousal: insights from electrophysiology, functional neuroimaging and psychopharmacology. Progress in Neurobiology.

[ref-20] Critchley HD, Mathias CJ, Dolan RJ (2001). Neural activity in the human brain relating to uncertainty and arousal during anticipation. Neuron.

[ref-21] Csikszentmihalyi M (1975). Beyond boredom and anxiety.

[ref-22] Csikszentmihalyi M, Pope JL, Singer kS (1978). Attention and the holistic approach to behavior. The Stream of Consciousness. Emotions, Personality, and Psychotherapy.

[ref-23] Csikszentmihalyi M (1990). Flow: the psychology of optimal experience.

[ref-24] Csikszentmihalyi M (2014). Flow and the foundations of positive psychology.

[ref-26] Csikszentmihalyi M, Nakamura J (2010). Effortless attention in everyday life: a systematic phenomenology. Effortless Attention: A New Perspective in the Cognitive Science of Attention and Action.

[ref-27] de Manzano Ö, Cervenka S, Jucaite A, Hellenäs O, Farde L, Ullén F (2013). Individual differences in the proneness to have flow experiences are linked to dopamine D2-receptor availability in the dorsal striatum. NeuroImage.

[ref-28] de Manzano Ö, Theorell T, Harmat L, Ullén F (2010). The psychophysiology of flow during piano playing. Emotion.

[ref-29] de Sampaio Barros MF, Araújo-Moreira FM, Trevelin LC, Radel R (2018). Flow experience and the mobilization of attentional resources. Cognitive, Affective and Behavioral Neuroscience.

[ref-30] Delorme A, Makeig S (2004). EEGLAB: an open source toolbox for analysis of single-trial EEG dynamics including independent component analysis. Journal of Neuroscience Methods.

[ref-31] Dietrich A (2003). Functional neuroanatomy of altered states of consciousness: the transient hypofrontality hypothesis. Consciousness and Cognition.

[ref-32] Doufesh H, Ibrahim F, Ismail NA, Wan Ahmad WA (2014). Effect of muslim prayer (salat) on α electroencephalography and its relationship with autonomic nervous system activity. Journal of Alternative and Complementary Medicine.

[ref-33] Duncan J (2013). The structure of cognition: attentional episodes in mind and brain. Neuron.

[ref-34] Dunn PK, Smyth GK (1996). Randomized quantile residuals. Journal of Computational and Graphical Statistics.

[ref-35] Eckert MA, Menon V, Walczak A, Ahlstrom J, Denslow S, Horwitz A, Dubno JR (2009). At the heart of the ventral attention system: the right anterior insula. Human Brain Mapping.

[ref-36] Engelbregt HJ, Brinkman K, van Geest CCE, Irrmischer M, Deijen JB (2022). The effects of autonomous sensory meridian response (ASMR) on mood, attention, heart rate, skin conductance and EEG in healthy young adults. Experimental Brain Research.

[ref-37] Eom TH (2023). Electroencephalography source localization. Clinical and Experimental Pediatrics.

[ref-38] Filippini C, Di Crosta A, Palumbo R, Perpetuini D, Cardone D, Ceccato I, Di Domenico A, Merla A (2022). Automated affective computing based on bio-signals analysis and deep learning approach. Sensors.

[ref-39] Fornito A, Zalesky A, Bullmore E (2016). Fundamentals of brain network analysis.

[ref-40] Franken IHA, Zijlstra C, Muris P (2006). Are nonpharmacological induced rewards related to anhedonia? A study among skydivers. Progress in Neuro-Psychopharmacology and Biological Psychiatry.

[ref-41] Gaudreau P, Sanchez X, Blondin J-P (2006). Positive and negative affective states in a performance-related setting. European Journal of Psychological Assessment.

[ref-42] Gehan EA (1965). A generalized Wilcoxon test for comparing arbitrarily singly-censored samples. Biometrika.

[ref-43] German DC, Bowden DM (1974). Catecholamine systems as the neural substrate for intracranial self-stimulation: a hypothesis. Brain Research.

[ref-44] Gold J, Ciorciari J (2020). A review on the role of the neuroscience of flow states in the modern world. Behavioral Sciences.

[ref-45] Gold J, Ciorciari J (2021). A neurocognitive model of flow states and the role of cerebellar internal models. Behavioral Brain Research.

[ref-46] Goodale MA, Milner AD (1992). Separate visual pathways for perception and action. Trends in Neurosciences.

[ref-47] Hailstone J, Kilding AE (2011). Reliability and validity of the Zephyr^TM^ BioHarness^TM^ to measure respiratory responses to exercise. Measurement in Physical Education and Exercise Science.

[ref-61] Hänsel A, von Känel R (2008). The ventro-medial prefrontal cortex: a major link between the autonomic nervous system, regulation of emotion, and stress reactivity?. BioPsychoSocial Medicine.

[ref-48] Hare OA, Wetherell MA, Smith MA (2013). State anxiety and cortisol reactivity to skydiving in novice versus experienced skydivers. Physiology & Behavior.

[ref-49] Harmat L, de Manzano Ö, Theorell T, Högman L, Fischer H, Ullén F (2015). Physiological correlates of the flow experience during computer game playing. International Journal of Psychophysiology.

[ref-50] Harris DJ, Vine SJ, Wilson MR (2017a). Flow and quiet eye: the role of attentional control in flow experience. Cognitive Processing.

[ref-51] Harris DJ, Vine SJ, Wilson MR (2017b). Neurocognitive mechanisms of the flow state. Progress in Brain Research.

[ref-52] Harris DJ, Vine SJ, Wilson MR, Harris DJ, Vine SJ, Wilson MR (2016). Is flow really effortless? The complex role of effortful attention. Sport, Exercise, and Performance Psychology.

[ref-53] Harrison BJ, Fullana MA, Via E, Soriano-Mas C, Vervliet B, Martínez-Zalacaín I, Pujol J, Davey CG, Kircher T, Straube B, Cardoner N (2017). Human ventromedial prefrontal cortex and the positive affective processing of safety signals. NeuroImage.

[ref-54] Hellige JB (1993). Hemispheric asymmetry: what’s right and what’s left.

[ref-55] Hermans EJ, Henckens MJAG, Joëls M, Fernández G (2014). Dynamic adaptation of large-scale brain networks in response to acute stressors. Trends in Neurosciences.

[ref-56] Holmbom M, Brymer E, Schweitzer RD (2017). Transformations through proximity flying: a phenomenological investigation. Frontiers in Psychology.

[ref-57] Hunter J, Csikszentmihalyi M (2000). The phenomenology of body-mind: the contrasting cases of flow in sports and contemplation. Anthropology of Consciousness.

[ref-58] Huskey R, Wilcox S, Weber R (2018). Network neuroscience reveals distinct neuromarkers of flow during media use. Journal of Communication.

[ref-59] Huxley A (1956). Heaven and hell.

[ref-60] Hyvärinen A, Oja E (2000). Independent component analysis: algorithms and applications. Neural Networks.

[ref-62] Immonen T, Brymer E, Orth D, Davids K, Feletti F, Liukkonen J, Jaakkola T (2017). Understanding action and adventure sports participation—an ecological dynamics perspective. Sports Medicine-Open.

[ref-67] Jäncke L, Leipold S, Burkhard A (2018). The neural underpinnings of music listening under different attention conditions. NeuroReport.

[ref-63] Jackson SA, Csikszentmihalyi M (1999). Flow in sports.

[ref-64] Jackson SA, Eklund RC (2004). The flow scales manual.

[ref-65] Jackson SA, Martin AJ, Eklund RC (2008). Long and short measures of flow: the construct validity of the FSS-2, DFS-2, and new brief counterparts. Journal of Sport and Exercise Psychology.

[ref-66] Japee S, Holiday K, Satyshur MD, Mukai I, Ungerleider LG (2015). A role of right middle frontal gyrus in reorienting of attention: a case study. Frontiers in Systems Neuroscience.

[ref-68] Kawabata M, Mallett CJ (2011). Flow experience in physical activity: examination of the internal structure of flow from a process-related perspective. Motivation and Emotion.

[ref-69] Kerr JH, Houge Mackenzie S (2012). Multiple motives for participating in adventure sports. Psychology of Sport and Exercise.

[ref-70] Kim JH, Roberge R, Powell JB, Shafer AB, Jon Williams W (2013). Measurement accuracy of heart rate and respiratory rate during graded exercise and sustained exercise in the heat using the zephyr BioHarness TM. International Journal of Sports Medicine.

[ref-71] Koessler L, Maillard L, Benhadid A, Vignal JP, Felblinger J, Vespignani H, Braun M (2009). Automated cortical projection of EEG sensors: anatomical correlation via the international 10-10 system. NeuroImage.

[ref-72] Kotler S, Mannino M, Kelso S, Huskey R (2022). First few seconds for flow: a comprehensive proposal of the neurobiology and neurodynamics of state onset. Neuroscience & Biobehavioral Reviews.

[ref-73] Lee SY, Kim SM, Lee RS, Park IR (2024). Effect of participation motivation in sports climbing on leisure satisfaction and physical self-efficacy. Behavioral Sciences.

[ref-74] Lehmann D, Faber PL, Tei S, Pascual-Marqui RD, Milz P, Kochi K (2012). Reduced functional connectivity between cortical sources in five meditation traditions detected with lagged coherence using EEG tomography. NeuroImage.

[ref-75] Leroy A, Cheron G (2020). EEG dynamics and neural generators of psychological flow during one tightrope performance. Scientific Reports.

[ref-76] Lim J, Wu WC, Wang J, Detre JA, Dinges DF, Rao H (2010). Imaging brain fatigue from sustained mental workload: an ASL perfusion study of the time-on-task effect. NeuroImage.

[ref-77] Ludwig KA, Miriani RM, Langhals NB, Joseph MD, Anderson DJ, Kipke DR (2009). Using a common average reference to improve cortical neuron recordings from microelectrode arrays. Journal of Neurophysiology.

[ref-78] Luo C, Li F, Li P, Yi C, Li C, Tao Q, Zhang X, Si Y, Yao D, Yin G, Song P, Wang H, Xu P (2022). A survey of brain network analysis by electroencephalographic signals. Cognitive Neurodynamics.

[ref-79] Madras BK (2013). History of the discovery of the antipsychotic dopamine D2 receptor: a basis for the dopamine hypothesis of schizophrenia. Journal of the History of the Neurosciences.

[ref-81] Mańkowska A, Harciarek M, Williamson JB, Heilman KM (2018). The influence of rightward and leftward spatial deviations of spatial attention on emotional picture recognition. Journal of Clinical and Experimental Neuropsychology.

[ref-80] Martin JM, Altarriba J (2017). Effects of valence on hemispheric specialization for emotion word processing. Language and Speech.

[ref-82] Milner AD, Goodale MA (2008). Two visual systems re-viewed. Neuropsychologia.

[ref-83] Moen F, Myhre K, Rasdal V, Vittersø J, Sandbakk Ø (2017). The role of emotions for 4 athletes in Nordic combined in ski jumping competitions in World Cup. 19, March. https://thesportjournal.org/article/the-role-of-emotions-for-4-athletes-in-nordic-combined-in-ski-jjumping-competitions-in-world-cup/.

[ref-84] Monasterio E, Mei-Dan O, Hackney AC, Lane AR, Zwir I, Rozsa S, Cloninger CR (2016). Stress reactivity and personality in extreme sport athletes: the psychobiology of BASE jumpers. Physiology & Behavior.

[ref-85] Montull L, Vázquez P, Rocas L, Hristovski R, Balagué N (2020). Flow as an embodied state. Informed awareness of slackline walking. Frontiers in Psychology.

[ref-86] Mujica-Parodi LR, Carlson JM, Cha J, Rubin D (2014). The fine line between ‘brave’ and ‘reckless’: amygdala reactivity and regulation predict recognition of risk. NeuroImage.

[ref-87] Norman J (2002). Two visual systems and two theories of perception: an attempt to reconcile the constructivist and ecological approaches. Behavioral and Brain Sciences.

[ref-88] Nyberg L, Cabeza R (2000). Imaging cognition II: an empirical review of 275 PET and fMRI studies. Journal of Cognitive Neuroscience.

[ref-89] Ogg RJ, Zou P, Allen DN, Hutchins SB, Dutkiewicz RM, Mulhern RK (2008). Neural correlates of a clinical continuous performance test. Magnetic Resonance Imaging.

[ref-90] Pain MTG, Pain MA (2005). Essay: risk taking in sport. Lancet (London, England).

[ref-91] Partington S, Partington E, Olivier S (2009). The dark side of flow: a qualitative study of dependence in big wave surfing. The Sport Psychologist.

[ref-92] Paus T, Zatorre RJ, Hofle N, Caramanos Z, Gotman J, Petrides M, Evans AC (1997). Time-related changes in neural systems underlying attention and arousal during the performance of an auditory vigilance task. Journal of Cognitive Neuroscience.

[ref-93] Peifer C, Schulz A, Schächinger H, Baumann N, Antoni CH (2014). The relation of flow-experience and physiological arousal under stress—can u shape it?. Journal of Experimental Social Psychology.

[ref-94] Pels F, Kleinert J, Mennigen F (2018). Group flow: a scoping review of definitions, theoretical approaches, measures and findings. PLOS ONE.

[ref-95] Pessoa L (2017). A network model of the emotional brain. Trends in Cognitive Sciences.

[ref-96] Pessoa L (2018). Understanding emotion with brain networks. Current Opinion in Behavioral Sciences.

[ref-25] Polechoński J, Szczechowicz B, Ryśnik J, Tomik R (2024). Recreational cycling provides greater satisfaction and flow in an immersive virtual environment than in real life. BMC Sports Science, Medicine and Rehabilitation.

[ref-97] Pizam A, Reichel A, Uriely N (2001). Sensation seeking and tourist behavior. Journal of Hospitality & Leisure Marketing.

[ref-201] R Core Team (2019). R: a Language and Environment for Statistical Computing.

[ref-98] Rigby RA, Stasinopoulos DM (2005). Generalized additive models for location, scale and shape. Journal of the Royal Statistical Society. Series C: Applied Statistics.

[ref-99] Rogers BP, Morgan VL, Newton AT, Gore JC (2007). Assessing functional connectivity in the human brain by fMRI. Magnetic Resonance Imaging.

[ref-100] Rojas GM, Alvarez C, Montoya CE, de la Iglesia-Vayá M, Cisternas JE, Gálvez M (2018). Study of resting-state functional connectivity networks using EEG electrodes position as seed. Frontiers in Neuroscience.

[ref-101] Rufi S, Wlodarczyk A, Páez D, Javaloy F (2016). Flow and emotional experience in spirituality: differences in interactive and coactive collective rituals. Journal of Humanistic Psychology.

[ref-102] Schall JD (2004). On the role of frontal eye field in guiding attention and saccades. Vision Research.

[ref-103] Scott-Hamilton J, Schutte NS, Brown RF (2016). Effects of a mindfulness intervention on sports-anxiety, pessimism, and flow in competitive cyclists. Applied Psychology: Health and Well-Being.

[ref-104] Seidel-Marzi O, Hähner S, Ragert P, Carius D (2021). Task-related hemodynamic response alterations during slacklining: an fnirs study in advanced slackliners. Frontiers in Neuroergonomics.

[ref-105] Shiota MN, Neufeld SL, Yeung WH, Moser SE, Perea EF (2011). Feeling good: autonomic nervous system responding in five positive emotions. Emotion.

[ref-106] Siegel M, Donner TH, Engel AK (2012). Spectral fingerprints of large-scale neuronal interactions. Nature Reviews Neuroscience.

[ref-107] Sparks JR (2016). Extreme sports: a study of free-solo rock climbers. Master thesis.

[ref-108] Srinivasan R, Winter WR, Ding J, Nunez PL (2007). EEG and MEG coherence: measures of functional connectivity at distinct spatial scales of neocortical dynamics. Journal of Neuroscience Methods.

[ref-109] Stein K, Mombaur K (2022). A quantitative comparison of slackline balancing capabilities of experts and beginners. Frontiers in Sports and Active Living.

[ref-110] Sterlini GL, Bryant RA (2002). Hyperarousal and dissociation: a study of novice skydivers. Behaviour Research and Therapy.

[ref-111] Thompson ER (2007). Development and validation of an internationally reliable short-form of the positive and negative affect schedule (PANAS). Journal of Cross-Cultural Psychology.

[ref-112] Tozman T, Magdas ES, MacDougall HG, Vollmeyer R (2015). Understanding the psychophysiology of flow: a driving simulator experiment to investigate the relationship between flow and heart rate variability. Computers in Human Behavior.

[ref-113] Ueta K, Mizuguchi N, Sugiyama T, Isaka T, Otomo S (2022). The motor engram of functional connectivity generated by acute whole-body dynamic balance training. Medicine and Science in Sports and Exercise.

[ref-114] Ullén F, De Manzano Ö, Theorell T, Harmat L (2010). The physiology of effortless attention: correlates of state flow and flow proneness. Effortless Attention: A New Perspective in the Cognitive Science of Attention and Action.

[ref-115] Walz JM, Goldman RI, Carapezza M, Muraskin J, Brown TR, Sajda P (2013). Simultaneous EEG-fMRI reveals temporal evolution of coupling between supramodal cortical attention networks and the brainstem. Journal of Neuroscience.

[ref-116] Weber R, Fisher JT (2020). The roots of the flow construct.

[ref-117] Weber R, Tamborini R, Westcott-Baker A, Kantor B (2009). Theorizing flow and media enjoyment as cognitive synchronization of attentional and reward networks. Communication Theory.

[ref-118] Welch PD (1975). The use of fast fourier transform for the estimation of power spectra. Digital Signal Processing.

[ref-119] Wiersma LD (2014). A phenomenological investigation of the psychology of big-wave surfing at Maverick’s. Sport Psychologist.

[ref-120] Woodman T, Huggins M, Le Scanff C, Cazenave N (2009). Alexithymia determines the anxiety experienced in skydiving. Journal of Affective Disorders.

[ref-121] Yu D, Wang S, Song F, Liu Y, Zhang S, Wang Y, Zhang Z (2023). Research on user experience of the video game difficulty based on flow theory and fNIRS. Behavior & Information Technology.

[ref-122] Yuksel A, Olmez T, Renda M, Bursa M, Holzinger A, Khuri S (2016). Filter bank common spatio-spectral patterns for motor imagery classification. Information Technology in Bio- and Medical Informatics. ITBAM 2016. Lecture Notes in Computer Science..

